# Molecular Epidemiology of Coxsackievirus A16: Intratype and Prevalent Intertype Recombination Identified

**DOI:** 10.1371/journal.pone.0082861

**Published:** 2013-12-10

**Authors:** Xiangpeng Chen, Xiaojuan Tan, Jing Li, Yu Jin, Liming Gong, Mei Hong, Yonglin Shi, Shuangli Zhu, Baomin Zhang, Shuang Zhang, Yong Zhang, Naiying Mao, Wenbo Xu

**Affiliations:** 1 Key Laboratory of Medical Virology Ministry of Health, National Institute for Viral Disease Control and Prevention, Chinese Center for Disease Control and Prevention, Beijing, P. R. of China; 2 Yangzhou No.1 People’s Hospital, Yangzhou, P. R. of China; 3 Nanjing Children’s Hospital, Nanjing Medical University, Nanjing, P. R. of China; 4 Zhejiang Provincial Center for Disease Control and Prevention, Hangzhou, P. R. of China; 5 Xizang Provincial Center for Disease Control and Prevention, Lasa, P. R. of China; 6 Anhui Provincial Center for Disease Control and Prevention, Hefei, P. R. of China; 7 Shunyi Center for Disease Control and Prevention, Beijing, P. R. of China; University of Hong Kong, Hong Kong

## Abstract

Coxsackievirus A16 (CVA16) is responsible for nearly 50% of all the conﬁrmed hand, foot, and mouth disease (HFMD) cases in mainland China, sometimes it could also cause severe complications, and even death. To clarify the genetic characteristics and the epidemic patterns of CVA16 in mainland China, comprehensive bioinfomatics analyses were performed by using 35 CVA16 whole genome sequences from 1998 to 2011, 593 complete CVA16 *VP1* sequences from 1981 to 2011, and prototype strains of human enterovirus species A (EV-A). Analysis on complete *VP1* sequences revealed that subgenotypes B1a and B1b were prevalent strains and have been co-circulating in many Asian countries since 2000, especially in mainland China for at least 13 years. While the prevalence of subgenotype B1c (totally 20 strains) was much limited, only found in Malaysia from 2005 to 2007 and in France in 2010. Genotype B2 only caused epidemic in Japan and Malaysia from 1981 to 2000. Both subgenotypes B1a and B1b were potential recombinant viruses containing sequences from other EV-A donors in the 5’-untranslated region and P2, P3 non-structural protein encoding regions.

## Introduction

In recent years, the hand, foot and mouth disease (HFMD) has been a very common infection for children in Asian countries caused by various enteroviruses, with coxsackievirus A16 (CVA16) and enterovirus 71 (EV-A71) as the two major causative agents [1]. Since EV-A71 was more frequently associated with severe complications including aseptic meningitis, acute ﬂaccid paralysis, brainstem encephalitis, pulmonary edema, and even death, so there were a lot of studies focusing on EV-A71 [2-5], but relatively less on CVA16. Studies on molecular epidemiology of CVA16 were reported elsewhere [6-10], the genetic diversity and evolutionary characteristics of this virus has yet to be explored. Previous studies have shown that CVA16 was responsible for the conﬁrmed HFMD cases in China in recent years [11-13]; besides HFMD and herpangina, infection by CVA16 could also cause serious myocarditis and pericarditis, acute heart failure, and even death [14,15].

The genome of CVA16 is a single stranded, positive sense RNA, containing approximately 7,410 nucleotides with a single open-reading-frame (ORF). The viral genome contains 5'- and 3’- untranslated regions (UTRs) which are essential for viral RNA replication and expression. The 5’-UTR is about 740 nucleotides in length and comprises the internal ribosomal entry site, which is related to the replication and internal initiation of translation of the genomic RNA [16]. The ORF has 6,579 nucleotides, encoding a polyprotein of 2,193 amino acids which is composed of 3 protein precursors: P1, P2, and P3. The P1 polyprotein precursor processed into 4 structural proteins, VP1 to VP4, while P2 and P3 are precursors of the seven nonstructural proteins; 2A, 2B, 2C, 3A, 3B, 3C, and 3D, respectively [17,18]. 

Since CVA16 was ﬁrst identiﬁed in 1951 in South Africa, it evolved slowly [19]. Complete genomic analysis of Enterovirus A (EV-A) indicated that recombination in the nonstructural region played an important role in the evolution of some EV-A strains [20]. Recombination between CVA16 and other serotypes of EV-A in the nonstructural region had also been documented previously by using partial sequences available earlier [11], however, the recombination analysis was also limited by the lack of complete genome sequences. To clarify the global molecular epidemiology and genetic characteristic of CVA16, we perform an extensive genetic analysis using all of the available genomic sequences of CVA16 dated before December 2012 in the public database, plus 8 complete genome sequences determined in our laboratory.

## Results

### Genotyping based on complete VP1 sequences of CVA16

**Figure 1:**
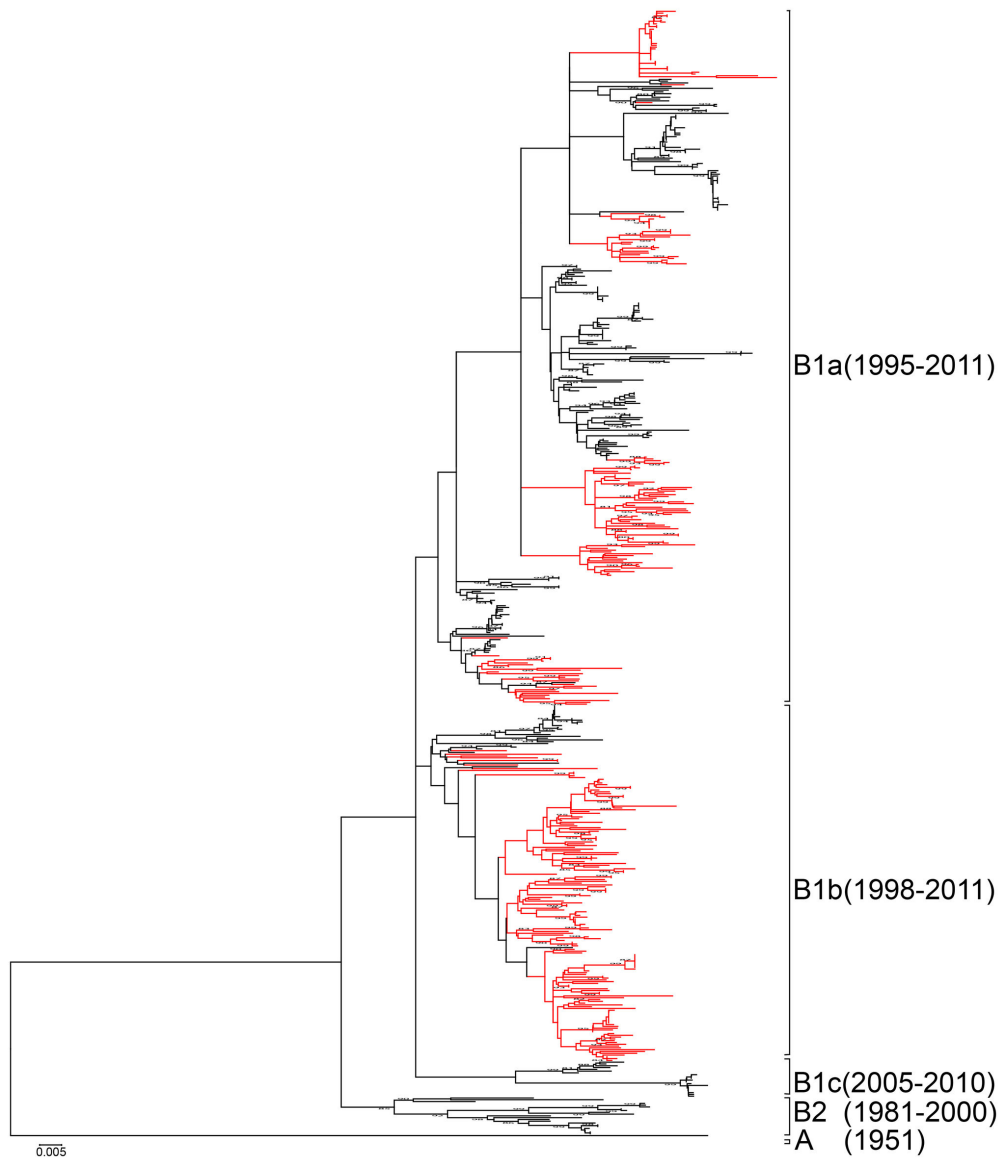
The Neighbor-joining tree of all the 629 CVA16 viruses based on complete VP1 encoding sequences. The branches showed in red color highlight the sequences from China.

A total of 629 sequences with complete *VP1* region of CVA 16 were used for analysis, included 621 retrieved from public database dated before December 2012, plus 8 complete genome sequences determined by our laboratory (Table 1, Table S2). Phylogenetic analyses were performed with MEGA software. CVA16 strains could be divided into genotypes A, B1 and B2 as indicated in our previous study [19]. Further, genotype B1 could be divided into subgenotypes B1a, B1b, and B1c. Among 629 *VP1* sequences, 607 clustered together with the reference sequence of genotype B1, and 21 showed the nearest relationship with genotype B2, while the prototype CVA16 was the unique member of genotype A (Figure 1). The genotype B2 was first reported from Japan in 1981 (AB465366). Among the 21 B2 strains, 17 were isolated from Japan during 1981-1998, and 4 from Malaysia during 1998-2000. After 2000, there was no genotype B2 reported. The earliest B1 strains were isolated in 1995 from Japan (AB634295-AB634311). Relatively, genotype B1 was more prevalent in the epidemics and had been reported in many countries, including mainland China, Taiwan, Japan, Malaysia, Thailand, Vietnam, Australia, France, Korea, Spain, Sweden and Saudi Arabia since then. 

**Table 1:** List of the complete genomes sequences of 35 CVA16 strains used for the sequence comparison, phylogenetic and recombination analysis.

Strain name	Source	GenBank Accession Number	The place of isolation	The year of isolation	genotype
Kor08/South Korea/2008	GenBank	JX839965	South Korea	2008	B1a
PM-12284-99/MAL/1999	GenBank	JQ746661	Malaysia	1999	B1a
PM-15765-00/MAL/2000	GenBank	JQ746666	Malaysia	2000	B1a
PM-00033-07/MAL/2007	GenBank	JQ746660	Malaysia	2007	B1a
THA-CA16-090/Thailand/2010	GenBank	JF738003	Thailand	2010	B1a
THA-CA16-069/Thailand/2010	GenBank	JF738004	Thailand	2010	B1a
Tainan/5079/98/Taiwan/1998	GenBank	AF177911	Taiwan	1998	B1a
BJ/11/11/BJ/CHN/2011	GenBank	JX068831	Beijing, China	2011	B1a
KMM/08/CHN/2008	GenBank	HQ423141	Yunnan, China	2008	B1a
TS10/08/CHN/2010	GenBank	JX068829	China	2010	B1a
SZ/HK08-3/HK/CHN/2008	GenBank	GQ279368	HongKong, China	2008	B1a
Ningbo.CHN/028-2/2009/ZJ/CHN/2009	GenBank	JQ354992	Zhejiang, China	2009	B1a
GZ08/GD/CHN/2008	GenBank	FJ198212	Guangdong, China	2008	B1b
HQ09011181/YN/CHN/2011	GenBank	JQ316639	Yunnan, China	2011	B1a
shzh05-1/GD/CHN/2005	GenBank	EU262658	Guangdong, China	2005	B1b
TS10/07/CHN/2010	GenBank	JX068827	China	2010	B1b
BJ09/06/BJ/CHN/2009	GenBank	JX068832	Beijing, China	2009	B1b
BJCA08/BJ/CHN/2008	GenBank	JX481738	Beijing, China	2008	B1b
BJ08/07/BJ/CHN/2008	GenBank	JX068833	Beijing, China	2008	B1b
BJ11/03/BJ/CHN/2011	GenBank	JX068830	Beijing, China	2011	B1b
BJ11/12/BJ/CHN/2011	GenBank	JX068828	Beijing, China	2011	B1b
HN1662/HeN/CHN/2010	GenBank	JN674176	Henan, China	2010	B1b
G20/YN/CHN/2010	GenBank	JN590244	Yunnan, China	2010	B1b
XM-CA16-3560/FJ/CHN/2009	GenBank	HQ269389	Fujian, China	2009	B1b
shzh00-1/GD/CHN/2000	GenBank	AY790926	Guangdong, China	2000	B1b
SZ/HK08-7/HK/CHN/2008	GenBank	GQ279371	HongKong, China	2008	B1b
SH/CHN/2009/ CHN/2010	GenBank	JQ034149	Shanghai, China	2010	B1b
ZJ10-48	This study	KC755235	Zhejiang, China	2010	B1a
ZJ10-73	This study	KC755229	Zhejiang, China	2010	B1a
AH10-2	This study	KC755230	Anhui, China	2010	B1a
XZ10-C-1	This study	KC755234	Tibet, China	2010	B1a
NJ10-31	This study	KC755232	Jiangsu, China	2010	B1a
XZ10-D-1	This study	KC755228	Tibet, China	2010	B1b
NJ10-75	This study	KC755233	Jiangsu, China	2010	B1b
AH10-12	This study	KC755231	Anhui, China	2010	B1b

**Figure 2:**
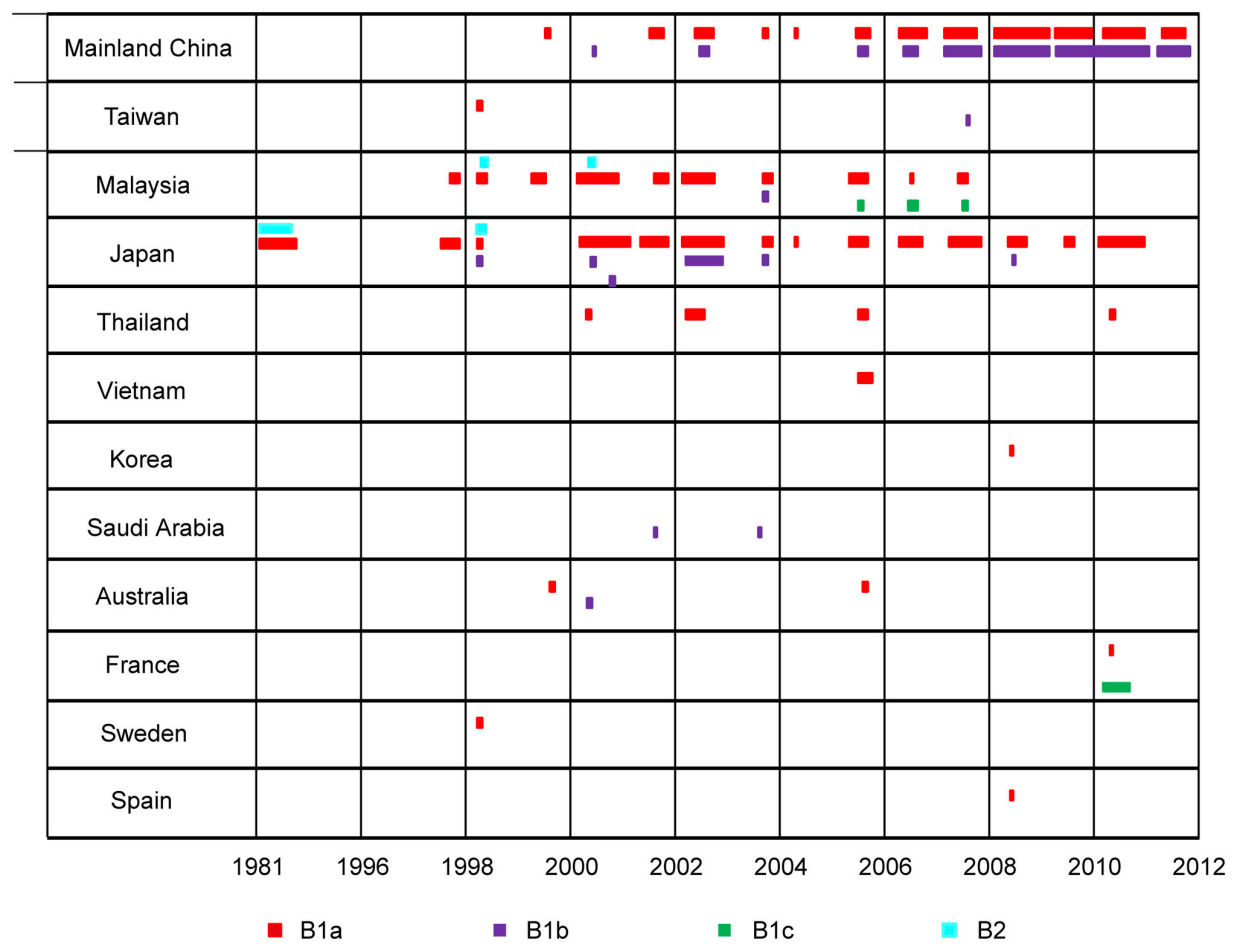
Global distribution of subgenotypes of CVA16. The time and spatial distribution of all genotypes, except genotype A, were shown in this chart. All the 628 genotype B strains from all other countries were shown in this chart except CVA16 prototype strain. Bar in blue, red, purple, and green indicate the subgenotype B2, B1a, B1b, and B1c, respectively. The length of the bar correlated with the numbers of the isolates in corresponding year.

Among 607 genotype B1 strains, there were 387, 200, and 20 strains could be divided into subgenotypes B1a, B1b, and B1c, respectively, by phylogenetic analysis (Figure 1). In more detail, B1a was the most prevalent one, after its first appearance in 1995 in Japan, it has been circulating in 11 of the 12 countries (regions) involved in this study, except Saudi Arabia. B1b was reported in mainland China, Taiwan, Japan, Malaysia, Australia, and Saudi Arabia during 1998-2011. Circulation of B1c was only detected in Malaysia from 2005-2007, and in France in 2010 (Figure 2).

In our study, most of the sequence data (582/629, 92.5%) were reported from Japan, Malaysia, and China. In these three countries, B1a (357/582, 61%) was the most reported genotype, which has been persistently circulating for more than 15 years (Figure 2). In mainland China, B1b was another dominant genotype (169/293, 58%) which has been co-circulating with B1a (124/293, 42%) since 2000. In Malaysia, besides B1a, B1c was also consecutively identified during 2005-2007. While in Japan, although B1b had co-circulated with B1a during 1998-2004, B1a has become the predominant genotype after 2004 (Figure 2).

### Analysis of complete genomic sequences of CVA16

For further genomic analysis, a total of 35 complete genome sequences of CVA16 were used, including one prototype, 8 from our laboratory, and 27 retrieved from public database. Among the 35 genome sequences, there were 26 sequences from mainland China, 3 from Malaysia, 2 each from Hong Kong and Thailand, and 1 each from South Korea and Taiwan (Table 1). Phylogenetically, these 35 isolates were grouped into subgenotype B1a (18) and B1b (17) based on complete *VP1* region, and all of the B1a sequences were from China (Figure 1, Figure S1a). 

Compared to the CVA16 prototype (U05876), there were no deletions or insertions observed in the P1, P2, and P3 encoding regions. Nucleotide substitutions among different CVA16 strains are scattered throughout the genome. Pairwise nucleotide sequence identities are 89.4%-98.9% among the 35 isolates, 91.4%-98.5% and 93.2-98.9% within groups B1a and B1b, respectively. Pairwise nucleotide sequence identities based on each gene were also calculated (Table S4). In the structural capsid protein encoding regions, VP1-VP4, nucleotide sequence identities within subgenotype B1a and B1b are more than 91.3% and 92.2%, respectively. Comparatively, sequences in the non-structural protein encoding regions showed more diversities, especially in 3B (Table S4), the least identity in which is 81.8%. Estimations of mean genetic distance also indicated high homology in the whole genome, which were 0.049 within B1a, 0.043 within B1b (Table 2). The mean nucleotide distance between B1a and B1b is 0.086, which is significantly larger than mean genetic distance within groups. The mean amino acid distance is much smaller, which was 0.009 within both B1a and B1b, and 0.013 between B1a and B1b (Table 2). Both the values in sequence identity and genetic distance suggested a near phylogenetic relationship of sequences within both B1a and B1b.

**Table 2:** Mean genetic distances based on corresponding regions of 35 complete genomic sequences and amino acid sequences of CVA16.

Region	distance			
	B1a	B1b	B1	Between B1a and B1b
	nt aa	nt aa	nt aa	nt aa
complete	0.049 0.009	0.043 0.009	0.066 0.011	0.086 0.013
5UTR*	0.037 --	0.030 --	0.045 --	0 .050 --
P1	0.048 0.005	0.044 0.005	0.066 0.005	0.086 0.005
VP4	0.047 0.005	0.030 0.012	0.054 0.008	0.069 0.008
VP2	0.056 0.009	0.045 0.003	0.070 0.006	0.088 0.006
VP3	0.045 0.001	0.049 0.005	0.070 0.003	0.092 0.003
VP1	0.045 0.004	0.042 0.004	0.063 0.004	0.092 0.004
P2	0.050 0.008	0.040 0.008	0.066 0.010	0.085 0.012
2A	0.056 0.017	0.039 0.017	0.039 0.020	0.088 0.022
2B	0.048 0.011	0.042 0.011	0.059 0.011	0.072 0.012
2C	0.047 0.003	0.041 0.003	0.066 0.005	0.087 0.008
P3	0.053 0.016	0.049 0.016	0.074 0.021	0.096 0.025
3A	0.052 0.015	0.048 0.018	0.077 0.031	0.103 0.045
3B	0.053 0.024	0.067 0.024	0.101 0.024	0.140 0.023
3C	0.060 0.019	0.045 0.010	0.077 0.016	0.099 0.018
3D	0.050 0.015	0.050 0.018	0.071 0.020	0.092 0.024

Note: Kimura-2-parameter nucleotide substitution model was used for estimation of mean genetic distance of the nucleotide sequences, and *p*-distance, which is the proportion (p) of amino acid sites at which the two sequences to be compared are different, for mean genetic distance of the amino acid sequences. *: Pairwise comparisons not including THA-CA16-090/Thailand/2010, THA-CA16-069/Thailand/2010, and HQ09011181/YN/CHN/2011 for partial missings in 5’UTR. Abbreviation: nt, nucleotide; aa, amino acid.

**Figure 3:**
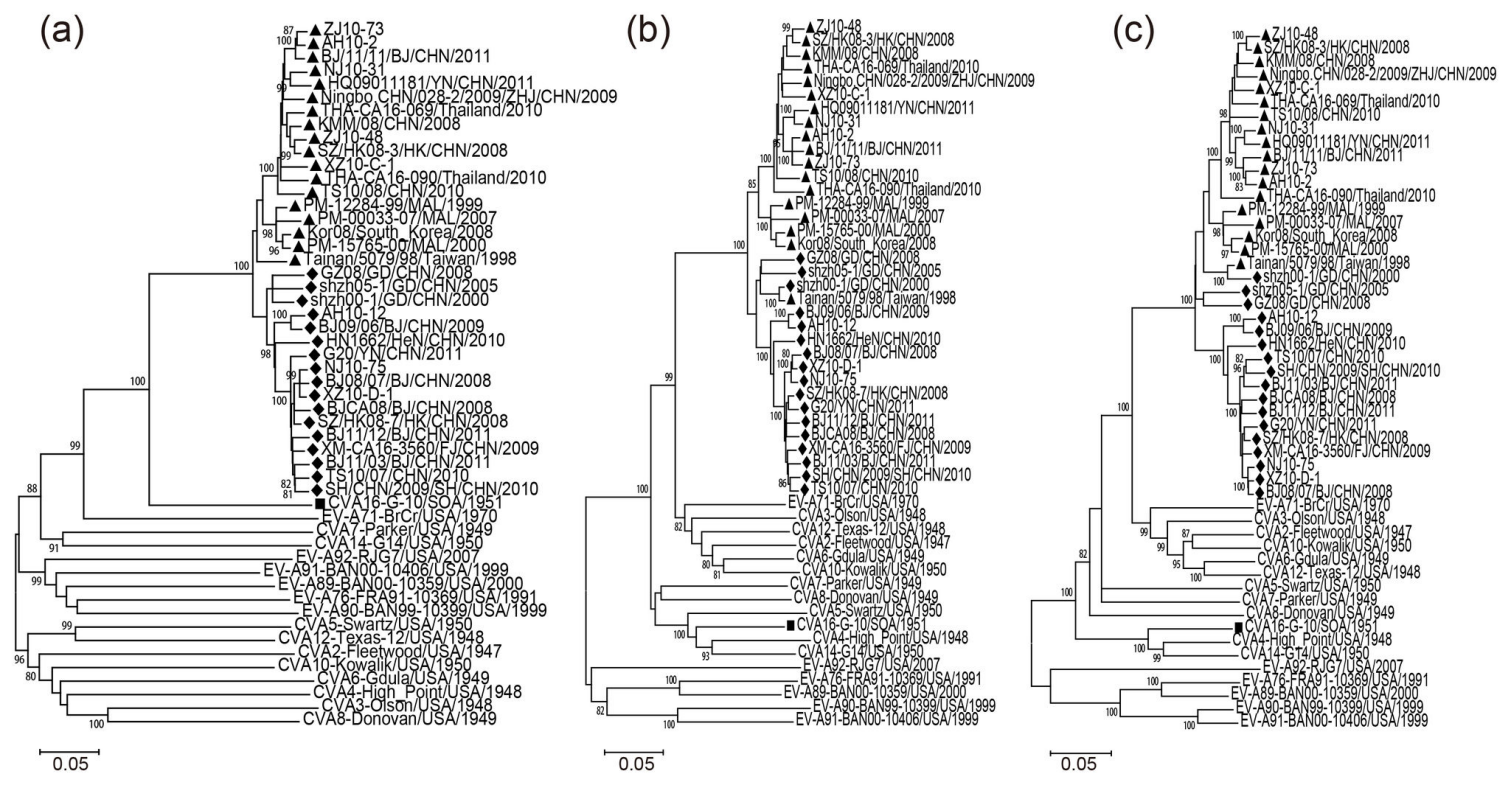
Phylogenetic dendrogram showing the relationships between the cluster B1a, B1b and EV-A prototypes. These were constructed by the different genomic regions of 35 complete CVA16 genomic sequences and EV-A prototype sequences. The neighbour-joining trees were reconstructed based on the P1 (a), P2 (b), P3 (c) genomic regions and complete genomes (d), respectively. Triangles indicated the subgenotype B1a. Rhombuses indicated the subgenotype B1b. Squares indicated the CVA16 prototype G-10 strain. The percentage of bootstrap (percentage of 1000 pseudoreplicate datasets) supporting the trees are indicated at the nodes; only values over 80% are shown.

Phylogenetic dendrograms based on the whole genomes, protein encoding regions, and each specific gene were performed with MEGA 5.0 software as shown in Figure 3 and Figure S1-2. The grouping of B1a and B1b in phylogenetic trees based on *P1*, *P2*, and *P3* regions were different (Figure 3), which suggested potential intratypic recombinations between B1a and B1b had occurred. This has also been confirmed by phylogenetic analyses on each specific gene (Figure S1-2). Several sequences of subgenotype B1a clustered with B1b on *VP2-VP4* (Figure S1), and the grouping on *2A-2C*, and *3A-3D* were more diverse (Figure S2). 

Moreover, a potential intertypic recombination between the 35 genomic sequences of CVA16 and other serotypes of EV-A was also observed. Based on nucleotide sequences of *P1* and its encoding proteins, all of the 35 sequences displayed nearest phylogenic relationship with CVA16 prototype (Figure 3a, Figure S1). However, based on other non-capsid protein encoding regions and *5’ UTR*, all sequences exhibited further distance in evolutionary origin with CVA16 than other EV-A serotypes. In dendrograms based on *P2* and *P3*, all 35 sequences clustered with a clade including the prototypes of EV-A71, CVA2, CVA3, CVA6, CVA10 and CVA12 but not CVA16 (Figures 3b, 3c). The different topology of the dendrograms between *P1* and *P2-P3* regions suggested a potential intertypic recombination of these circulating CVA16 strains with other EV-A serotypes in non-structural protein encoding regions. This deduction could also be supported by the nucleotide sequence identities with every prototype of EV-A. In both *P1* and mature proteins VP1-VP4 encoding regions, sequence identities with CVA16 prototype were 76.5%-78.0%, 75.1%-77.1%, 75.1%-78.8%, 76.7%-79.2% and 79.2%-83.5%, respectively (Table 3), these values are consistent with the 75% rule of enterovirus typing [21]. But in *P2* and *P3* regions, sequences in our study showed significantly higher homology to EV-A71, CVA2, CVA3, CVA6, CVA10 and CVA12, ranging from 81.1%-84.3% and 81.6%-84.1%, than to CVA16 and other prototypes, which were 79.4%-80.3% and 76.9%-78.1%, respectively (Table 3).

**Table 3:** Pairwise nucleotide sequences identities between 35 genomic sequences and prototype strains of EV-A species.

Regions	Identity (%)																
	G-10	EV-A71	CVA2	CVA3	CVA4	CVA5	CVA6	CVA7	CVA8	CVA10	CVA12	CVA14	EV-A76	EV-A89	EV-A90	EV-A91	EV-A92
Genome	77.3-79.1	76.7-79.1	74.3-76.1	74.5-76.4	71.8-73.7	72.6-74.8	74.2-76.0	74.1-75.8	72.6-74.5	74.5-76.5	74.7-76.6	72.5-74.5	68.2-69.3	68.8-70.1	67.2-68.6	68.0-69.7	66.6-68.0
5UTR	74.2-86.5	72.4-83.9	71.5-82.3	74.7-85.7	75.5-87.7	72.6-83.9	73.7-86.2	73.3-84.0	70.9-82.6	72.4-84.1	73.6-85.5	73.9-86.3	67.7-78.4	67.1-78.0	60.5-70.3	61.2-71.1	58.8-69.3
P1	76.5-78.0	69.0-69.9	62.8-64.1	61.7-62.8	61.5-62.6	63.2-64.4	61.6-62.2	64.9-66.0	62.8-63.7	64.3-65.3	64.1-65.1	65.5-66.3	62.8-64	63.8-65.1	62.8-63.5	64.8-65.8	63.1-64.4
VP4	79.2-83.5	69.0-72.4	63.2-67.1	62.8-65.7	64.2-66.6	60.3-63.7	62.3-65.2	62.8-65.7	59.9-62.8	62.8-66.6	61.3-64.7	60.3-63.7	63.7-66.6	61.8-64.2	64.2-67.6	66.1-70.0	60.3-63.7
VP2	75.1-78.8	70.6-72.7	64.5-66.4	64.7-66.5	65.6-67.3	65.7-67.5	65.4-68.3	68.5-70.7	65.0-67.4	66.9-69.2	68.7-70.8	68.9-70.5	64.8-66.5	64.8-66.6	63.7-66.1	66.2-68.4	66.0-68.3
VP3	76.7-79.2	70.3-72.3	66.3-69.0	63.3-65.2	63.3-65.4	66.7-68.9	64.3-66.5	67.7-69.9	64.7-66.5	65.2-67.3	65.6-67.6	68.1-70.1	65.9-68.5	68.0-70.3	69.2-70.9	67.7-70.5	66.0-68.8
VP1	75.1-77.1	64.5-66.3	57.0-59.2	56.7-58.3	55.2-56.8	55.6-57.0	53.8-56.0	58.2-59.5	59.0-60.4	59.8-61.4	56.1-57.9	59.7-62.0	58.2-59.5	60.9-62.6	57.9-59.7	60.5-62.5	58.3-60.8
P2	79.4-80.3	82.6-84.3	81.5-83.3	81.4-83.4	77.3-78.7	77.9-79.1	81.4-83.3	79.4-80.6	78.3-80.1	81.1-82.8	81.9-83.7	78.4-79.8	70.3-72.1	71.3-73.1	70.1-71.2	71.1-72.4	70.9-72.2
2A	79.1-81.7	78.2-81.1	78.4-81.1	79.3-82.8	75.1-78.4	79.5-82.6	78.2-80.4	76.0-79.7	75.7-80.4	79.3-82.0	77.7-81.1	77.3-81.1	64.2-66.4	68.0-71.3	62.0-64.8	64.6-68.0	64.6-67.1
2B	75.7-78.7	80.8-85.1	79.7-83.1	79.7-82.8	75.0-78.1	75.0-78.7	80.8-84.5	76.0-78.4	77.1-79.7	80.4-84.1	80.8-83.5	76.0-79.1	62.2-67.3	63.2-67.0	69.0-72.0	65.9-70.3	70.7-73.4
2C	79.6-81.3	84.4-86.5	82.7-85.5	82.7-84.4	78.0-79.9	76.6-78.8	82.0-85.3	81.0-82.5	78.9-80.9	81.1-83.6	83.2-86.0	78.7-80.7	75.2-76.7	74.8-76.4	73.4-74.5	74.7-76.0	72.8-74.7
P3	76.9-78.1	82.2-84.1	82.2-83.7	82.5-83.7	76.4-78.6	78.7-80.9	82.2-83.9	79.2-81.3	77.7-79.3	81.7-83.3	81.6-83.4	76.4-77.5	71.2-73.0	72.2-73.6	71.8-73.2	71.1-72.9	70.1-71.5
3A	79.4-81.7	83.3-87.9	80.2-86.4	81.7-85.2	77.9-81.3	75.9-79.4	81.7-86.4	77.9-81.0	76.3-79.8	81.0-86.0	81.7-86.8	77.1-81.3	67.8-73.2	68.9-73.6	68.6-72.0	67.8-72.0	62.7-67.0
3B	71.2-81.8	77.2-87.8	80.3-87.8	80.3-89.3	74.2-83.3	68.1-74.2	80.3-89.3	66.6-75.7	69.6-77.2	78.7-86.3	78.7-87.8	71.2-80.3	68.1-75.7	66.6-75.7	60.6-68.1	59.0-68.1	60.6-72.7
3C	73.4-76.6	82.6-84.8	81.2-83.2	81.9-84.6	75.0-77.9	76.1-78.8	81.2-83.2	76.6-81.0	75.5-77.2	80.1-82.6	81.2-84.3	74.4-77.0	70.6-72.6	72.1-74.1	69.5-73.0	71.0-73.7	69.0-73.0
3D	76.9-78.4	81.3-84.0	81.7-84.3	81.8-83.9	76.1-78.7	80.3-83.1	81.6-84.3	80.1-82.3	78.4-80.8	81.6-84.0	80.7-83.6	75.9-77.5	71.3-73.8	72.1-74.0	72.7-74.5	71.6-73.8	71.2-72.8

Phylogenetic trees from each of mature protein encoding regions of *P2* (*2A-2C*) and *P3* (*3A-3D*), indicated more details of this inconsistency. Based on each specific region of *P2* and *P3*, all the sequences in our study clustered along with the same clade in *P2* and *P3*, except in *2A*. In the later case, sequences in our study still showed nearest phylogenetic relationship with the prototype of CVA16, but indicating a low bootstrap value (Figure S2a). These data suggested a heterogenous origin of *2B*, *2C*, *3A*, *3B*, *3C* and *3D* in all 35 sequences (Figure 3, Figure S2). Calculated values of nucleotide sequence identities in the *2B*, *2C*, *3A*, *3B*, *3C* and *3D* regions also showed consistency with phylogenetic dendrograms. While in *2A* region, the sequence identities showed no significant difference between CVA16 and others, suggesting a multi-source in *2A* of our sequences (Table 3), and possibly the site which intertypic recombination occurred. 

**Figure 4:**
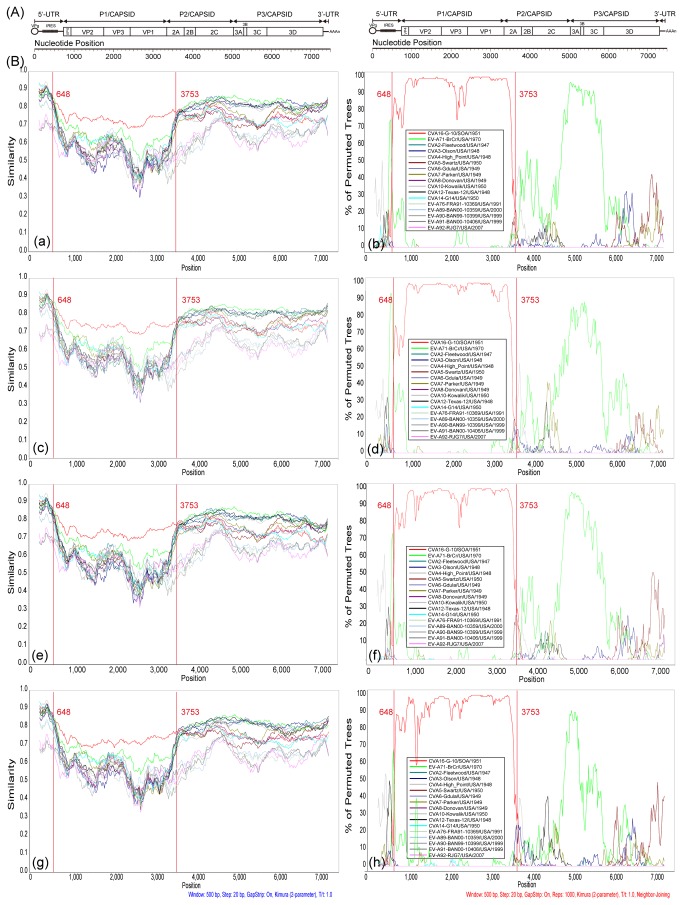
Similarity plot and bootscanning analyses of all the two subgenotypes B1a, B1b of CVA16 and other EV-A prototype strains on the basis of full-length genomes. Two strains were chosen as the representative strains of each subgenotype, respectively. Each of the four viruses was used as the query sequence. A sliding window of 500 nucleotides moving in 20 nucleotides steps was used in this analysis. (a and b) Tainan/5079/98/Taiwan/1998; (c and d) BJ/11/11 /BJ/CHN/2011; (e and f) shzh00-1/GD/CHN/2000; (g and h) BJ11/12/BJ/CHN/2011. The a, c, e, g were the results of similarity plot analyses; the b, d, f, h were the results of bootscanning analyses.

To further depict the possible recombination of these strains, similarity plot and bootscanning analyses were performed by using EV-A prototype strains as reference sequences. Four strains, Tainan/5079/98/Taiwan/1998, shzh00-1/GD/CHN/2000, BJ/11/11/BJ/CHN/2011, and BJ/11/12/BJ/CHN/2011, were chosen as the representative strains. These represented the oldest and the latest CVA16 strains of subgenotypes B1a and B1b, respectively (Figure 4). As indicated in the plots, these 4 sequences showed definitely the highest similarity and the nearest phylogenetic relationship with CVA16 prototype in *P1* region. However, in *P2* and *P3* regions, all 4 strains contained some unidentified regions that showed a little higher similarity with prototypes of EV-A71, CVA2, CVA3, CVA6, CVA10 and CVA12, but apparently not CVA16 (Figures 4a, 4c, 4e, and 4g). The bootscanning showed a nearer phylogenetic relationship with EV-A71 (Figures 4b, 4d, 4f, and 4h). An obvious single crossing site in the *2A* region suggested a potential recombination occurred which is consistent with deduction above.

## Discussion

CVA16 was involved in a significantly high transmission among the children with HFMD and herpangina. The symptoms caused by EV-A71 and CVA16 are difficult to differentiate clinically. The co-circulation or alternating circulation of CVA16 and EV-A71 had been proven to have contributed to the outbreaks of HFMD [8,22,23]. CVA16 was responsible for about 50% of all the conﬁrmed enterovirus infections among HFMD cases in mainland China [8,24-26]. The infection by CVA16 was usually mild and benign when compared to EV-A71 infection, however, severe complications such as myocarditis and pericarditis, even death could occur [14,15].

There was lack of systematic epidemiologic analysis on CVA16 in previous studies. We studied the molecular epidemiology of CVA16 by analyzing all the complete *VP1* sequences from GenBank database, and clarified the global distribution of the CVA16 genotypes. All CVA16 strains belonged to the genotype B, except the CVA16 prototype, which was the unique strain in genotype A. From 1981 to 2000, majority of the CVA16 strains classified as genotype B2 were only detected in Japan and Malaysia. The subgenotypes B1a and B1b strains were first isolated in 1995 and 1998, respectively, in Japan. These two subgentypes then became the predominant ones circulating globally since 2000. In mainland China, all the CVA16 strains isolated belonged to subgenotypes B1a and B1b. Both subgenotypes could be detected in most of the provinces. However, only single subgenotype B1b was detected in Shanghai, Shaanxi, Jilin, Fujian, Tianjin and Inner Mongolia. There has no subgenotype B1c been detected in China, so the surveillance on this new subgenotype must be continually reinforced.

In this study, high nucleotide sequence identities were observed among all 35 CVA16 complete genome sequences and in their respective encoding regions. Phylogenetic analyses also indicated that these 35 CVA16 strains consistently clustered together in all of the analyzed regions. These result suggested that all 35 CVA16 sequences had high homogeneity with a little internal differences. This revealed that CVA16 strains might have evolved independently, steadily and originated from a same ancestor. It was determined that neutralization epitopes resided mainly on capsid proteins, especially protein VP1 [27]. In our results, the amino acid sequence identities in the *P1* and *VP1* regions of these 35 CVA16 strains were 99.5% and 99.6%, respectively. Further amino acid sequence comparisons revealed that the field epidemic CVA16 strains also had high sequence identities in protein P1 and detailed VP1-VP4 encoding regions, both within or between the subgenotypes B1a and B1b strains (Table 2). CVA16 immunity might cross-react with EV-A71 [28], however, the amino acid sequence identities in the P1 and VP1-VP4 regions were 79.3%, 71.9%, 84.2%, 85.4%, 78.1% between EV-A71 subgenotype C4 representative strain (JN256060) and prevalent CVA16 strains in our study (data not shown). So, the cross-react neutralization epitopes mighty reside on the proteins VP2 and VP3. The genetic stability in the structural proteins and simplex genotype transmission in China are very important for designing serological diagnostic test and vaccine to the prevalent CVA16 strains.

This study demonstrated that the current CVA16 strains were potential multiple-recombinant viruses containing other EV-A donor sequences. Recombination was frequently detected in the *P2* and *P3* regions while it was rare in *P1* region, probably because of the structural proteins was important for the virus replication and assembling of proper viral capsid [29-31]. Genetic recombination sometimes could result in the virulence change of enteroviruses, which could trigger serious public health problems [32]. Because the multiple intertypic recombinations occurred in the *P2* and *P3* regions long time before and only few complete sequences of CVA16 are available in GenBank database, so the phylogenetic analyses are limited and hard to determine the donor strains.

These isolated CVA16 strains from HFMD outbreaks in mainland China recently were the sustained circulation of the same B1a and B1b strains from a common ancestor without clearly new genetic recombination and with a slow evolutionary rate. To elucidate further detailed genetic characteristics and evolutionary recombination events in CVA16, continually strengthen on surveillance is necessary.

## Materials and Methods

### Ethics Statement

This study did not involve human experimentation or human participants; the only human materials used were stool, throat swab, and vesicles samples collected from HFMD patients at the instigation of the Ministry of Health P. R. of China (PRC) for public health purposes. HFMD is a notifiable disease in China, and the pathogenic surveillance of HFMD without private information referred is required by the Law of the PRC on the Prevention and Treatment of Infectious Diseases. According to this law, these data used in this study were obtained from samples as part of this monitoring program. Thus, the requirement for written informed consent was waived by the Ethics Review Committee of the National Institute for Viral Disease Control and Prevention. This study was approved by the Ethics Review Committee of the National Institute for Viral Disease Control and Prevention. The CVA16 isolates involved in this study were isolated from the clinical samples of HFMD patients in 2010. These patients are from different geographical locations in the Tibet Autonomous Region, Zhejiang, Jiangsu and Anhui provinces of China. 

### Viruses

In this study, eight CVA16 strains were isolated from eight HFMD patients in mainland China in 2010 respectively. Patients were children with the age range from 2 to 14 years old. Viruses were isolated from throat swabs, feces, and vesicle fluid specimens on human rhabdomyosarcoma (RD) cell line. 

### Determination of the genomes

The complete genome of the 8 CVA16 isolates mentioned above were determined in our laboratory. Viral RNA was extracted using a QIAamp Viral RNA Mini Kit (Qiagen, Valencia, CA, USA). First, the viral RNA was converted to cDNA by a random priming strategy. The cDNA was amplified using the primers designed by multiple alignments of CVA16 genomes available in GenBank database (Table S1). PCR products were purified for sequencing using the QIAquick Gel extraction kit (Qiagen). Then the amplicons were bi-directionally sequenced using fluorescent dideoxy terminators and an ABI PRISM 3100 genetic analyzer (Applied Biosystems Foster City, CA, USA). 

### Sequences from GenBank

Totally 593 complete *VP1* encoding sequences (Table S2) and 28 complete genome sequences (Table 1) of CVA16, which released to public database before December 2012, were downloaded from GenBank. The sequences of prototypes of EV-A group (CVA2, 3, 4, 5, 6, 7, 8, 10, 12, 14, EV-A71, 76, 89, 90, 91 and 92) were used as references in the phylogenetic and recombination analysis (Table S3). These 28 genome sequences of CVA16 that downloaded from GenBank database included 20 from mainland China and 8 from Malaysia (3), Thailand (2), Korea (1), Taiwan (1), and South Africa (1) from 1998 to 2011. 

### Bioinformatics and Phylogenetic analysis

Trimming of the sequencing chromatogram and sequences assembly were preformed with Sequencher program (version 4.0.5 Gene Codes Corporation, USA). Phylogenetic trees were reconstructed with MEGA program (version 5.0; Sudhir Kumar, Arizona State University, Tempe, Arizona, USA) [33], using the neighbor-joining method and Kimura-2-parameter substitution model. (Note: Maximum likelihood method has also been used to construct the phylogenetic trees to increases the reliability, the results were more or less the same as neighbor-joining method. Data not shown.) Bootstrap testing with 1000 replicates were used to estimate the strength of the phylogenetic trees. Bootstrap values greater than 80% were considered to be a strong support for the tree topology. Completely sequenced 28 CVA16 isolates available in the GenBank, 8 isolates from our laboratory and other EV-A prototype strains were aligned and used for construction of phylogenetic trees and recombiantion analyses. The genetic distance and identity matrices were determined by group mean and pairwise estimation of the sequences divergence with MEGA program [34]. 

### Recombination analysis

Alignment of the complete genome sequences were performed with the ClustalW package in MEGA program (version 5.0; Sudhir Kumar, Arizona State University, Tempe, Arizona, USA) [33]. Potential recombination between CVA16 and other EV-A strains was scanned by Similarity plot and bootscan method (version 3.5.1; Stuart Ray, Johns Hopkins University, Baltimore, Maryland, USA) [35]. The neighbor-joining method and Kimura 2-parameter substitution model were used. A sliding window size of 500bp nucleotides moving in 20bp nucleotides at a time was used in this analysis.

### Nucleotide sequence accession numbers

Complete genome sequences of the 8 CVA16 strains have been submitted to the GenBank database with the accession numbers KC755228 to KC755235.

## Supporting Information

Table S1 **List of nucleotide sequences of primer for amplification of the whole genome sequences of CVA16.**
(DOCX)

Table S2 **List of the 593 complete VP1 sequences of CVA16 strains available from GenBank which were selected to generate the CVA16 phylogenetic dendrograms.**
(DOCX)

Table S3 **List of the EV-A prototype strains used for the sequence comparison, phylogenetic and recombination analysis.**
(DOCX)

Table S4 **Pairwise nucleotide sequence identities based on corresponding regions of 35 complete genomic sequences of CVA16.**
(DOCX)

Figure S1 **Phylogenetic dendrograms showing the relationships amongst EV-A isolates using the different genomic regions.** Phylogenetic dendrogram showing the relationships between the cluster B1a, B1b and EV-A prototypes by using the different genomic regions of 35 complete CVA16 genomic sequences and EV-A prototype sequences. The neighbour-joining trees were constructed from alignment of the VP1 (a), VP2 (b), VP3 (c) and VP4 (d) genomic regions, respectively. Triangles indicated the subgenotype B1a. Rhombuses indicated the subgenotype B1b. Squares indicated the CVA16 prototype G-10 strain. The percentage of bootstrap (percentage of 1000 pseudoreplicate datasets) replicates supporting the trees are indicated at the nodes; for clarity, only values over 80% are shown. The branch lengths are proportional to the genetic distances corrected using Kimura-two-parameter substitution model.
(EPS)

Figure S2 **Phylogenetic dendrograms showing the relationships amongst EV-A isolates using the different genomic regions.** Phylogenetic dendrogram showing the relationships between the cluster B1a, B1b and EV-A prototypes by using the different genomic regions of 35 complete CVA16 genomic sequences and EV-A prototype sequences. The neighbour-joining trees were constructed from alignment of the 2A (a), 2B (b), 2C (c), 3A (d), 3B (e), 3C (f), 3D (g) and 5’UTR (h) genomic regions, respectively. Triangles indicated the subgenotype B1a. Rhombuses indicated the subgenotype B1b. Squares indicated the CVA16 prototype G-10 strain. The percentage of bootstrap (percentage of 1000 pseudoreplicate datasets) replicates supporting the trees are indicated at the nodes; for clarity, only values over 80% are shown. The branch lengths are proportional to the genetic distances corrected using Kimura-two-parameter substitution model.
(EPS)
