# Supplementary material for: Molecular Epidemiology of Coxsackievirus A16: Intratype and Prevalent Intertype Recombination Identified
Source: PLoS One. 2013 Dec 10;8(12):e82861. doi: 10.1371/journal.pone.0082861 (PMC3858299; doi:10.1371/journal.pone.0082861)
Supplement: Table S1 — List of nucleotide sequences of primer for amplification of the whole genome sequences of CVA16. (DOCX) [file pone.0082861.s001.docx]

**TABLE S1. List of nucleotide sequences of primer for amplification of the whole genome sequences of CVA16**

| Primer | Nucleotide position (nt) | Primer sequence (5’-3’) | Orientation | Reference |
| --- | --- | --- | --- | --- |
| 0001S48a |  | GGGGACAAGTTTGTACAAAAAAGCAGGCTTTAAAACAGCTCTGGGGTT | Forward | [[1](#_ENREF_1)] |
| CVA16-596Z | 577-596 | ACACGGACACCCAAAGTAGTCGGTTC | Reverse | This study |
| EV/PCR-2 | 449-473 | TCCGGCCCCTGAATGCGGCTAATCC | Forward | [[2](#_ENREF_2)] |
| CVA16-1254Z | 1235-1254 | TGGAACTGAGCGTTTTGACC | Reverse | This study |
| CVA16-1058Y | 1058-1077 | GCCTATGGAGAGTGGCCTGA | Forward | This study |
| CVA16-1983Z | 1964-1983 | GCCCTAAAGGCAGCACATAA | Reverse | This study |
| CVA16-1790Y | 1790-1809 | ATTCTGCCAGGTTTCCATCC | Forward | This study |
| CVA16-2800Z | 2781-2800 | CTGGGCATATCCCATCAAGT | Reverse | This study |
| CVA16-VP1-S | 2335-2354 | ATTGGTGCTCCCACTACAGC | Forward | [[3](#_ENREF_3)] |
| CVA16-VP1-A | 3426-3445 | GCTGTCCTCCCACACAAGAT | Reverse | [[3](#_ENREF_3)] |
| CVA16-3292Y | 3292-3311 | GACATCAAATGCACCAGCAC | Forward | This study |
| CVA16-4440Z | 4421-4440 | CGGTGTTTGCTCTTGAACTG | Reverse | This study |
| CVA16-4286Y | 4286-4305 | GCCTCGCAAGAGGATTTAGA | Forward | This study |
| CVA16-5400Z | 5381-5400 | GGTCCTTGGACTGTGGCTGT | Reverse | This study |
| CVA16-5204Y | 5204-5223 | CCAGAAACACCGACCAATGT | Forward | This study |
| CVA16-6157Z | 6138-6157 | GTCAGGCTCGTGTAGGGTGT | Reverse | This study |
| CVA16-5990Y | 5990-6009 | AACATTAATGGCCCAACTCG | Forward | This study |
| CVA16-6984Z | 6965-6984 | GTTTTGGCTAACTCCGAGCA | Reverse | This study |
| CVA16-6787Y | 6787-6806 | TTGTGTTCTTGGTGGAATGC | Forward | This study |
| 7500Aa |  | GGGGACCACTTTGTACAAGAAAGCTGGG(T)24 | Reverse | [[1](#_ENREF_1)] |

1. Yang CF, Naguib T, Yang SJ, Nasr E, Jorba J, et al. (2003) Circulation of endemic type 2 vaccine-derived poliovirus in Egypt from 1983 to 1993. J Virol 77: 8366-8377.

2. Yang CF, De L, Yang SJ, Ruiz Gomez J, Cruz JR, et al. (1992) Genotype-specific in vitro amplification of sequences of the wild type 3 polioviruses from Mexico and Guatemala. Virus Res 24: 277-296.

3. Zhang Y, Wang D, Yan D, Zhu S, Liu J, et al. (2010) Molecular evidence of persistent epidemic and evolution of subgenotype B1 coxsackievirus A16-associated hand, foot, and mouth disease in China. J Clin Microbiol 48: 619-622.
