# Supplementary material for: Molecular Epidemiology of Coxsackievirus A16: Intratype and Prevalent Intertype Recombination Identified
Source: PLoS One. 2013 Dec 10;8(12):e82861. doi: 10.1371/journal.pone.0082861 (PMC3858299; doi:10.1371/journal.pone.0082861)
Supplement: Table S2 — List of the 593 complete VP1 sequences of CVA16 strains available from GenBank which were selected to generate the CVA16 phylogenetic dendrograms. (DOCX) [file pone.0082861.s002.docx]

| **Table S2. List of the 593 complete VP1 sequences of CVA16 strains available from GenBank which were selected to generate the CVA16 phylogenetic dendrograms.** | | | | |
| --- | --- | --- | --- | --- |
| Strain name | Genbank Accession Number | The year of isolation | The place of isolation | genotype |
| 0001/AUS/99/AUS/1999 | AM292434 | 1999 | Australia | B1a |
| Y95-2096/JPN/1995 | AB634295 | 1995 | Japan | B1a |
| Y95-2447/JPN/1995 | AB634296 | 1995 | Japan | B1a |
| Y95-2135/JPN/1995 | AB634297 | 1995 | Japan | B1a |
| Y95-2142/JPN/1995 | AB634298 | 1995 | Japan | B1a |
| Y95-2164/JPN/1995 | AB634299 | 1995 | Japan | B1a |
| Y95-2200/JPN/1995 | AB634300 | 1995 | Japan | B1a |
| Y95-2259/JPN/1995 | AB634301 | 1995 | Japan | B1a |
| Y95-2260/JPN/1995 | AB634302 | 1995 | Japan | B1a |
| Y95-2313/JPN/1995 | AB634303 | 1995 | Japan | B1a |
| Y95-2318/JPN/1995 | AB634304 | 1995 | Japan | B1a |
| Y95-2345/JPN/1995 | AB634305 | 1995 | Japan | B1a |
| Y95-2386/JPN/1995 | AB634306 | 1995 | Japan | B1a |
| Y95-2390/JPN/1995 | AB634307 | 1995 | Japan | B1a |
| Y95-2403/JPN/1995 | AB634308 | 1995 | Japan | B1a |
| Y95-2854/JPN/1995 | AB634309 | 1995 | Japan | B1a |
| Y95-2874/JPN/1995 | AB634310 | 1995 | Japan | B1a |
| Y95-2932/JPN/1995 | AB634311 | 1995 | Japan | B1a |
| Y97-861/JPN/1997 | AB634312 | 1997 | Japan | B1a |
| Y97-1087/JPN/1997 | AB634313 | 1997 | Japan | B1a |
| Y97-1135/JPN/1997 | AB634314 | 1997 | Japan | B1a |
| Y97-1174/JPN/1997 | AB634315 | 1997 | Japan | B1a |
| Y97-1448/JPN/1997 | AB634316 | 1997 | Japan | B1a |
| 451-Yamagata-1997/JPN/1997 | AB634317 | 1997 | Japan | B1a |
| 494-Yamagata-1997/ JPN/1997 | AB634318 | 1997 | Japan | B1a |
| 496-Yamagata-1997/JPN/1997 | AB634319 | 1997 | Japan | B1a |
| MY823-3/SAR/97/MAL/1997 | AM292433 | 1997 | Malaysia | B1a |
| PM-14660-97/MAL/1997 | JN248408 | 1997 | Malaysia | B1a |
| PM-13884-97/MAL/1997 | JN248409 | 1997 | Malaysia | B1a |
| PM-14194-97/MAL/1997 | JN248410 | 1997 | Malaysia | B1a |
| 927/Toyama/1998/JPN/1998 | AB465371 | 1998 | Japan | B1a |
| S10051/SAR/98/MAL/1998 | AM292454 | 1998 | Malaysia | B1a |
| UM16809/MAL/98 | AM292483 | 1998 | Malaysia | B1a |
| PM-16809-98/MAL/1998 | JN248411 | 1998 | Malaysia | B1a |
| PM-16985-98/MAL/1998 | JN248412 | 1998 | Malaysia | B1a |
| 98-06281-35-01/Sweden/1998 | AY208085 | 1998 | Sweden | B1a |
| 99.132.2264/AUS/1999 | GU142869 | 1999 | Australia | B1a |
| shzh99-83/GD/CHN/1999 | AY821797 | 1999 | Guangdong,China | B1a |
| UM12593/MAL/99 | AM292478 | 1999 | Malaysia | B1a |
| UM12969/MAL/99 | AM292479 | 1999 | Malaysia | B1a |
| PM-12593-99/MAL/1999 | JN248404 | 1999 | Malaysia | B1a |
| PM-12284-99/MAL/1999 | JN248405 | 1999 | Malaysia | B1a |
| PM-12727-99/MAL/1999 | JN248406 | 1999 | Malaysia | B1a |
| PM-12969-99/MAL/1999 | JN248407 | 1999 | Malaysia | B1a |
| 624-Yamagata-2000/JPN/2000 | AB634325 | 2000 | Japan | B1a |
| 648-Yamagata-2000/JPN/2000 | AB634326 | 2000 | Japan | B1a |
| 649-Yamagata-2000/JPN/2000 | AB634327 | 2000 | Japan | B1a |
| 743-Yamagata-2000/JPN/2000 | AB634328 | 2000 | Japan | B1a |
| 744-Yamagata-2000/JPN/2000 | AB634329 | 2000 | Japan | B1a |
| 754-Yamagata-2000/JPN/2000 | AB634330 | 2000 | Japan | B1a |
| 755-Yamagata-2000/JPN/2000 | AB634331 | 2000 | Japan | B1a |
| 778-Yamagata-2000/JPN/2000 | AB634332 | 2000 | Japan | B1a |
| 780-Yamagata-2000/JPN/2000 | AB634333 | 2000 | Japan | B1a |
| 821-Yamagata-2000/JPN/2000 | AB634334 | 2000 | Japan | B1a |
| 826-Yamagata-2000/JPN/2000 | AB634335 | 2000 | Japan | B1a |
| 878-Yamagata-2000/JPN/2000 | AB634336 | 2000 | Japan | B1a |
| 1024-Yamagata-2000/JPN/2000 | AB634337 | 2000 | Japan | B1a |
| 787-Yamagata-2000/JPN/2000 | AB634338 | 2000 | Japan | B1a |
| 820-Yamagata-2000/JPN/2000 | AB634339 | 2000 | Japan | B1a |
| 835-Yamagata-2000/JPN/2000 | AB634340 | 2000 | Japan | B1a |
| 836-Yamagata-2000/JPN/2000 | AB634341 | 2000 | Japan | B1a |
| 904-Yamagata-2000/JPN/2000 | AB634342 | 2000 | Japan | B1a |
| 905-Yamagata-2000/JPN/2000 | AB634343 | 2000 | Japan | B1a |
| 984-Yamagata-2000/JPN/2000 | AB634344 | 2000 | Japan | B1a |
| 985-Yamagata-2000/JPN/2000 | AB634345 | 2000 | Japan | B1a |
| 988-Yamagata-2000/JPN/2000 | AB634346 | 2000 | Japan | B1a |
| 1050-Yamagata-2000/JPN/2000 | AB634347 | 2000 | Japan | B1a |
| 1069-Yamagata-2000/JPN/2000 | AB634348 | 2000 | Japan | B1a |
| 933-Yamagata-2000/JPN/2000 | AB634349 | 2000 | Japan | B1a |
| 939-Yamagata-2000/JPN/2000 | AB634350 | 2000 | Japan | B1a |
| 1007-Yamagata-2000/JPN/2000 | AB634351 | 2000 | Japan | B1a |
| 995-Yamagata-2000/JPN/2000 | AB634352 | 2000 | Japan | B1a |
| 1026-Yamagata-2000/JPN/2000 | AB634353 | 2000 | Japan | B1a |
| 1027-Yamagata-2000/JPN/2000 | AB634354 | 2000 | Japan | B1a |
| 1049-Yamagata-2000/JPN/2000 | AB634355 | 2000 | Japan | B1a |
| 1071-Yamagata-2000/JPN/2000 | AB634356 | 2000 | Japan | B1a |
| 1060-Yamagata-2000/JPN/2000 | AB634357 | 2000 | Japan | B1a |
| 1085-Yamagata-2000/JPN/2000 | AB634358 | 2000 | Japan | B1a |
| 1090-Yamagata-2000/JPN/2000 | AB634359 | 2000 | Japan | B1a |
| 1111-Yamagata-2000/JPN/2000 | AB634360 | 2000 | Japan | B1a |
| 1129-Yamagata-2000/JPN/2000 | AB634361 | 2000 | Japan | B1a |
| 1203-Yamagata-2000/JPN/2000 | AB634362 | 2000 | Japan | B1a |
| CNS041893/SAR/00/MAL/2000 | AM292446 | 2000 | Malaysia | B1a |
| CNS041904/SAR/00/MAL/2000 | AM292447 | 2000 | Malaysia | B1a |
| CNS043111/SAR/00/MAL/2000 | AM292448 | 2000 | Malaysia | B1a |
| CNS045384/SAR/00/MAL/2000 | AM292449 | 2000 | Malaysia | B1a |
| SB2000/SAR/00/MAL/2000 | AM292466 | 2000 | Malaysia | B1a |
| SB2002/SAR/00/MAL/2000 | AM292467 | 2000 | Malaysia | B1a |
| SB3512/SAR/00/MAL/2000 | AM292469 | 2000 | Malaysia | B1a |
| SB12115/SAR/03/MAL/2003 | AM292473 | 2000 | Malaysia | B1a |
| UM15797/MAL/00 | AM292480 | 2000 | Malaysia | B1a |
| UM15923/MAL/00 | AM292481 | 2000 | Malaysia | B1a |
| UM15985/MAL/00 | AM292482 | 2000 | Malaysia | B1a |
| UM17115/MAL/00 | AM292484 | 2000 | Malaysia | B1a |
| PM-15771-00/MAL/2000 | JN248392 | 2000 | Malaysia | B1a |
| PM-13998-00/MAL/2000 | JN248396 | 2000 | Malaysia | B1a |
| PM-15922-00/MAL/2000 | JN248398 | 2000 | Malaysia | B1a |
| PM-15773-00/MAL/2000 | JN248399 | 2000 | Malaysia | B1a |
| PM-17368-00/MAL/2000 | JN248400 | 2000 | Malaysia | B1a |
| PM-17106-00/MAL/2000 | JN248401 | 2000 | Malaysia | B1a |
| PM-17264-00/MAL/2000 | JN248402 | 2000 | Malaysia | B1a |
| PM-15912-00/MAL/2000 | JN248403 | 2000 | Malaysia | B1a |
| PM-14200-00/MAL/2000 | JN248413 | 2000 | Malaysia | B1a |
| TS1-2000/THAI/00 | AM292477 | 2000 | Thailand | B1a |
| Siriraj12/TH/00 | GQ184129 | 2000 | Thailand | B1a |
| shzh01-17/GD/CHN/2001 | AY821794 | 2001 | Guangdong,China | B1a |
| shzh01-12/GD/CHN/2001 | AY895096 | 2001 | Guangdong,China | B1a |
| shzh01-77/GD/CHN/2001 | AY895097 | 2001 | Guangdong,China | B1a |
| shzh01-58/GD/CHN/2001 | AY895101 | 2001 | Guangdong,China | B1a |
| shzh01-26/GD/CHN/2001 | AY895103 | 2001 | Guangdong,China | B1a |
| shzh01-68/GD/CHN/2001 | AY895104 | 2001 | Guangdong,China | B1a |
| shzh01-66/GD/CHN/2001 | AY895105 | 2001 | Guangdong,China | B1a |
| shzh01-69/GD/CHN/2001 | AY895111 | 2001 | Guangdong,China | B1a |
| 1113-Yamagata-2001/JPN/2001 | AB634363 | 2001 | Japan | B1a |
| 1120-Yamagata-2001/JPN/2001 | AB634364 | 2001 | Japan | B1a |
| 1130-Yamagata-2001/JPN/2001 | AB634365 | 2001 | Japan | B1a |
| 1195-Yamagata-2001/JPN/2001 | AB634366 | 2001 | Japan | B1a |
| 1268-Yamagata-2001/JPN/2001 | AB634367 | 2001 | Japan | B1a |
| 1540-Yamagata-2001/JPN/2001 | AB634368 | 2001 | Japan | B1a |
| 1250-Yamagata-2001/JPN/2001 | AB634369 | 2001 | Japan | B1a |
| 1393-Yamagata-2001/JPN/2001 | AB634370 | 2001 | Japan | B1a |
| 1562-Yamagata-2001/JPN/2001 | AB634371 | 2001 | Japan | B1a |
| 1581-Yamagata-2001/JPN/2001 | AB634372 | 2001 | Japan | B1a |
| 1649-Yamagata-2001/JPN/2001 | AB634373 | 2001 | Japan | B1a |
| 2011-Yamagata-2001/JPN/2001 | AB634374 | 2001 | Japan | B1a |
| CNS11062/SAR/01/MAL/2001 | AM292444 | 2001 | Malaysia | B1a |
| S33071/SAR/01/MAL/2000 | AM292458 | 2001 | Malaysia | B1a |
| S33072/SAR/01/MAL/2001 | AM292459 | 2001 | Malaysia | B1a |
| Siriraj10/TH/01 | GQ184130 | 2001 | Thailand | B1a |
| shzh02-124/GD/CHN/2002 | AY895094 | 2002 | Guangdong,China | B1a |
| shzh02-12/GD/CHN/2002 | AY895098 | 2002 | Guangdong,China | B1a |
| shzh02-16/GD/CHN/2002 | AY895100 | 2002 | Guangdong,China | B1a |
| shzh02-11/GD/CHN/2002 | AY895106 | 2002 | Guangdong,China | B1a |
| shzh02-78/GD/CHN/2002 | AY895108 | 2002 | Guangdong,China | B1a |
| shzh02-36/GD/CHN/2002 | AY895109 | 2002 | Guangdong,China | B1a |
| 255/Toyama/2002/JPN/2002 | AB465385 | 2002 | Japan | B1a |
| 256/Toyama/2002/JPN/2002 | AB465386 | 2002 | Japan | B1a |
| 259/Toyama/2002/JPN/2002 | AB465388 | 2002 | Japan | B1a |
| 261/Toyama/2002/JPN/2002 | AB465390 | 2002 | Japan | B1a |
| 262/Toyama/2002/JPN/2002 | AB465391 | 2002 | Japan | B1a |
| 263/Toyama/2002/JPN/2002 | AB465392 | 2002 | Japan | B1a |
| 264/Toyama/2002/JPN/2002 | AB465393 | 2002 | Japan | B1a |
| 265/Toyama/2002/JPN/2002 | AB465394 | 2002 | Japan | B1a |
| 266/Toyama/2002/JPN/2002 | AB465395 | 2002 | Japan | B1a |
| 995-Yamagata-2002/JPN/2002 | AB634375 | 2002 | Japan | B1a |
| 1687-Yamagata-2002/JPN/2002 | AB634376 | 2002 | Japan | B1a |
| 1872-Yamagata-2002/JPN/2002 | AB634377 | 2002 | Japan | B1a |
| 1895-Yamagata-2002/JPN/2002 | AB634378 | 2002 | Japan | B1a |
| 2071-Yamagata-2002/JPN/2002 | AB634379 | 2002 | Japan | B1a |
| 1747-Yamagata-2002/JPN/2002 | AB634380 | 2002 | Japan | B1a |
| 2527-Yamagata-2002/JPN/2002 | AB634381 | 2002 | Japan | B1a |
| 2067-Yamagata-2002/JPN/2002 | AB634382 | 2002 | Japan | B1a |
| 2319-Yamagata-2002/JPN/2002 | AB634384 | 2002 | Japan | B1a |
| 2679-Yamagata-2002/JPN/2002 | AB634385 | 2002 | Japan | B1a |
| 2724-Yamagata-2002/JPN/2002 | AB634386 | 2002 | Japan | B1a |
| 3184-Yamagata-2002/JPN/2002 | AB634387 | 2002 | Japan | B1a |
| EV1-5-HUKM/MAL/02 | AM292452 | 2002 | Malaysia | B1a |
| EV4-5-HUKM/MAL/02 | AM292453 | 2002 | Malaysia | B1a |
| S22781/SAR/02/MAL/2002 | AM292456 | 2002 | Malaysia | B1a |
| S22852/SAR/02/MAL/2002 | AM292457 | 2002 | Malaysia | B1a |
| SB7605/SAR/02/MAL/2002 | AM292470 | 2002 | Malaysia | B1a |
| SB7606/SAR/02/MAL/2002 | AM292471 | 2002 | Malaysia | B1a |
| SB7883/SAR/02/MAL/2002 | AM292472 | 2002 | Malaysia | B1a |
| PM-22159-02/MAL/2002 | JN248387 | 2002 | Malaysia | B1a |
| PM-22217-02/MAL/2002 | JN248388 | 2002 | Malaysia | B1a |
| PM-22241-02/MAL/2002 | JN248389 | 2002 | Malaysia | B1a |
| PM-22264-02/MAL/2002 | JN248390 | 2002 | Malaysia | B1a |
| PM-22339-02/MAL/2002 | JN248391 | 2002 | Malaysia | B1a |
| PM-23208-02/MAL/2002 | JN248397 | 2002 | Malaysia | B1a |
| Siriraj01/TH/02 | GQ184131 | 2002 | Thailand | B1a |
| Siriraj07/TH/02 | GQ184132 | 2002 | Thailand | B1a |
| Siriraj18/TH/02 | GQ184133 | 2002 | Thailand | B1a |
| Siriraj19/TH/02 | GQ184134 | 2002 | Thailand | B1a |
| Siriraj24/TH/02 | GQ184135 | 2002 | Thailand | B1a |
| Siriraj26/TH/02 | GQ184136 | 2002 | Thailand | B1a |
| shzh03-10/GD/CHN/2003 | AY895095 | 2003 | Guangdong,China | B1a |
| 2633-Yamagata-2003/JPN/2003 | AB634388 | 2003 | Japan | B1a |
| 2870-Yamagata-2003/JPN/2003 | AB634389 | 2003 | Japan | B1a |
| 2929-Yamagata-2003/JPN/2003 | AB634390 | 2003 | Japan | B1a |
| CNS32874/SAR/03/MAL/2003 | AM292445 | 2003 | Malaysia | B1a |
| S33421/SAR/03/MAL/2003 | AM292460 | 2003 | Malaysia | B1a |
| SB13044/SAR/03/MAL/2003 | AM292475 | 2003 | Malaysia | B1a |
| shzh04-J52/GD/CHN/2004 | AY895093 | 2004 | Guangdong,China | B1a |
| 2065-Yamagata-2004/JPN/2004 | AB634391 | 2004 | Japan | B1a |
| 0033/AUS/05 | AM292435 | 2005 | Australia | B1a |
| 05.194.4135/AUS/2005 | FJ868280 | 2005 | Australia | B1a |
| JB140500001/GD/CHN/2005 | HM776219 | 2005 | Guangdong,China | B1a |
| JB140500035/GD/CHN/2005 | HM776220 | 2005 | Guangdong,China | B1a |
| JB140500060/GD/CHN/2005 | HM776223 | 2005 | Guangdong,China | B1a |
| JB140500066/GD/CHN/2005 | HM776224 | 2005 | Guangdong,China | B1a |
| JB140500084/GD/CHN/2005 | HM776226 | 2005 | Guangdong,China | B1a |
| JB140500091/GD/CHN/2005 | HM776227 | 2005 | Guangdong,China | B1a |
| 355/Toyama/2005/JPN/2005 | AB465401 | 2005 | Japan | B1a |
| 2000-Yamagata-2005/JPN/2005 | AB634392 | 2005 | Japan | B1a |
| 2188-Yamagata-2005/JPN/2005 | AB634393 | 2005 | Japan | B1a |
| 2222-Yamagata-2005/JPN/2005 | AB634394 | 2005 | Japan | B1a |
| 2437-Yamagata-2005/JPN/2005 | AB634395 | 2005 | Japan | B1a |
| 2443-Yamagata-2005/JPN/2005 | AB634396 | 2005 | Japan | B1a |
| 2441-Yamagata-2005/JPN/2005 | AB634397 | 2005 | Japan | B1a |
| CNS51082/SAR/05/MAL/2005 | AM292450 | 2005 | Malaysia | B1a |
| S114131/SAR/05/MAL/2005 | AM292463 | 2005 | Malaysia | B1a |
| S114371/SAR/05/MAL/2005 | AM292464 | 2005 | Malaysia | B1a |
| PM-31174-05/MAL/2005 | JN248394 | 2005 | Malaysia | B1a |
| PM-32798-05/MAL/2005 | JN248395 | 2005 | Malaysia | B1a |
| PM-31033-05/MAL/2005 | JN248415 | 2005 | Malaysia | B1a |
| PM-31376-05/MAL/2005 | JN248416 | 2005 | Malaysia | B1a |
| Siriraj02/TH/05 | GQ184137 | 2005 | Thailand | B1a |
| Siriraj04/TH/05 | GQ184138 | 2005 | Thailand | B1a |
| Siriraj06/TH/05 | GQ184139 | 2005 | Thailand | B1a |
| 521V/VNM/05 | AM292436 | 2005 | Vietnam | B1a |
| 535V/VNM/05 | AM292437 | 2005 | Vietnam | B1a |
| 546V/VNM/05 | AM292438 | 2005 | Vietnam | B1a |
| 576T/VNM/05 | AM292439 | 2005 | Vietnam | B1a |
| 577T/VNM/05 | AM292440 | 2005 | Vietnam | B1a |
| 1018T/VNM/05 | AM292441 | 2005 | Vietnam | B1a |
| Siriraj07/TH/05 | GQ184140 | 2005 | Thailand | B1a |
| JB140600022/GD/CHN/2006 | HM776230 | 2006 | Guangdong,China | B1a |
| JB140600030/GD/CHN/2006 | HM776231 | 2006 | Guangdong,China | B1a |
| JB140600036/GD/CHN/2006 | HM776233 | 2006 | Guangdong,China | B1a |
| JB140600041/GD/CHN/2006 | HM776234 | 2006 | Guangdong,China | B1a |
| JB140600053/GD/CHN/2006 | HM776236 | 2006 | Guangdong,China | B1a |
| JB140600055/GD/CHN/2006 | HM776237 | 2006 | Guangdong,China | B1a |
| JB140600061/GD/CHN/2006 | HM776238 | 2006 | Guangdong,China | B1a |
| JB140600065/GD/CHN/2006 | HM776239 | 2006 | Guangdong,China | B1a |
| JB140600068/CHN/2006 | HM776240 | 2006 | Guangdong,China | B1a |
| JB140600069/GD/CHN/2006 | HM776241 | 2006 | Guangdong,China | B1a |
| JB140600077/GD/CHN/2006 | HM776242 | 2006 | Guangdong,China | B1a |
| JB140600086/GD/CHN/2006 | HM776244 | 2006 | Guangdong,China | B1a |
| 418/Toyama/2006/JPN/2006 | AB465402 | 2006 | Japan | B1a |
| 419/Toyama/2006/JPN/2006 | AB465403 | 2006 | Japan | B1a |
| 2295-Yamagata-2006/JPN/2006 | AB634398 | 2006 | Japan | B1a |
| 2511-Yamagata-2006/JPN/2006 | AB634399 | 2006 | Japan | B1a |
| 2402-Yamagata-2006/JPN/2006 | AB634400 | 2006 | Japan | B1a |
| 2421-Yamagata-2006/JPN/2006 | AB634401 | 2006 | Japan | B1a |
| 2620-Yamagata-2006/JPN/2006 | AB634402 | 2006 | Japan | B1a |
| 2681-Yamagata-2006/JPN/2006 | AB634403 | 2006 | Japan | B1a |
| 2686-Yamagata-2006/JPN/2006 | AB634405 | 2006 | Japan | B1a |
| 3162-Yamagata-2006/JPN/2006 | AB634406 | 2006 | Japan | B1a |
| 3195-Yamagata-2006/JPN/2006 | AB634407 | 2006 | Japan | B1a |
| 3242-Yamagata-2006/JPN/2006 | AB634408 | 2006 | Japan | B1a |
| CNS68762/SAR/06/MAL/2006 | AM292451 | 2006 | Malaysia | B1a |
| GS001F/GS/CHN/2007 | GQ429230 | 2007 | Gansu,China | B1a |
| GS002F/GS/CHN/2007 | GQ429231 | 2007 | Gansu,China | B1a |
| GS003F/GS/CHN/2007 | GQ429232 | 2007 | Gansu,China | B1a |
| GS004F/GS/CHN/2007 | GQ429233 | 2007 | Gansu,China | B1a |
| GS005F/GS/CHN/2007 | GQ429234 | 2007 | Gansu,China | B1a |
| GS006F/GS/CHN/2007 | GQ429235 | 2007 | Gansu,China | B1a |
| GS007T/GS/CHN/2007 | GQ429236 | 2007 | Gansu,China | B1a |
| JB140700001/CHN/2007 | HM776246 | 2007 | Guangdong,China | B1a |
| JB140700008/GD/CHN/2007 | HM776247 | 2007 | Guangdong,China | B1a |
| JB140700016/CHN/2007 | HM776248 | 2007 | Guangdong,China | B1a |
| JB140700028/GD/CHN/2007 | HM776250 | 2007 | Guangdong,China | B1a |
| 459/Toyama/2007/JPN/2007 | AB465404 | 2007 | Japan | B1a |
| 460/Toyama/2007/JPN/2007 | AB465405 | 2007 | Japan | B1a |
| 584-Yamagata-2007/JPN/2007 | AB634404 | 2007 | Japan | B1a |
| 512-Yamagata-2007/JPN/2007 | AB634409 | 2007 | Japan | B1a |
| 1122-Yamagata-2007/JPN/2007 | AB634410 | 2007 | Japan | B1a |
| 1152-Yamagata-2007/JPN/2007 | AB634411 | 2007 | Japan | B1a |
| 1098-Yamagata-2007/JPN/2007 | AB634412 | 2007 | Japan | B1a |
| 1618-Yamagata-2007/JPN/2007 | AB634413 | 2007 | Japan | B1a |
| 1246-Yamagata-2007/JPN/2007 | AB634414 | 2007 | Japan | B1a |
| 1249-Yamagata-2007/JPN/2007 | AB634415 | 2007 | Japan | B1a |
| 2377-Yamagata-2007/JPN/2007 | AB634416 | 2007 | Japan | B1a |
| 1293-Yamagata-2007/JPN/2007 | AB634417 | 2007 | Japan | B1a |
| 1430-Yamagata-2007/JPN/2007 | AB634418 | 2007 | Japan | B1a |
| 1600-Yamagata-2007/JPN/2007 | AB634419 | 2007 | Japan | B1a |
| 1872-Yamagata-2007/JPN/2007 | AB634420 | 2007 | Japan | B1a |
| PM-1791021-07/MAL/2007 | JN248393 | 2007 | Malaysia | B1a |
| PM-00033-07/MAL/2007 | JN248414 | 2007 | Malaysia | B1a |
| 520-03F/SD/CHN/2007 | GQ429220 | 2007 | Shandong,China | B1a |
| 521-06F/SD/CHN/2007 | GQ429223 | 2007 | Shandong,China | B1a |
| 522-25F/SD/CHN/2007 | GQ429224 | 2007 | Shandong,China | B1a |
| BJ176/BJ/2008/CHN/2008 | JF317966 | 2008 | Beijing,China | B1a |
| GS0019T/GS/CHN/2008 | GQ429241 | 2008 | Gansu,China | B1a |
| GS0026F/GS/CHN/2008 | GQ429242 | 2008 | Gansu,China | B1a |
| GS0039V/GS/CHN/2008 | GQ429244 | 2008 | Gansu,China | B1a |
| GS0041V/GS/CHN/2008 | GQ429245 | 2008 | Gansu,China | B1a |
| GS0151V/GS/CHN/2008 | GQ429246 | 2008 | Gansu,China | B1a |
| GS0155T/GS/CHN/2008 | GQ429247 | 2008 | Gansu,China | B1a |
| GS0246F/GS/CHN/2008 | GQ429249 | 2008 | Gansu,China | B1a |
| GS0363F/GS/CHN/2008 | GQ429255 | 2008 | Gansu,China | B1a |
| GS0373F/GS/CHN/2008 | GQ429258 | 2008 | Gansu,China | B1a |
| GS0429V/GS/CHN/2008 | GQ429262 | 2008 | Gansu,China | B1a |
| GS0430F/GS/CHN/2008 | GQ429263 | 2008 | Gansu,China | B1a |
| JB14080004/GD/CHN/2008 | HM776252 | 2008 | Guangdong,China | B1a |
| JB14080019/GD/CHN/2008 | HM776253 | 2008 | Guangdong,China | B1a |
| JB14080041/GD/CHN/2008 | HM776256 | 2008 | Guangdong,China | B1a |
| JB14080100/GD/CHN/2008 | HM776258 | 2008 | Guangdong,China | B1a |
| JB14080165/GD/CHN/2008 | HM776260 | 2008 | Guangdong,China | B1a |
| JB14080183/GD/CHN/2008 | HM776261 | 2008 | Guangdong,China | B1a |
| JB14080302/GD/CHN/2008 | HM776262 | 2008 | Guangdong,China | B1a |
| JB14080331/GD/CHN/2008 | HM776263 | 2008 | Guangdong,China | B1a |
| JB14080342/GD/CHN/2008 | HM776264 | 2008 | Guangdong,China | B1a |
| JB14080389/GD/CHN/2008 | HM776265 | 2008 | Guangdong,China | B1a |
| JB14080413/GD/CHN/2008 | HM776266 | 2008 | Guangdong,China | B1a |
| JB14080437/GD/CHN/2008 | HM776267 | 2008 | Guangdong,China | B1a |
| JB14080485/GD/CHN/2008 | HM776268 | 2008 | Guangdong,China | B1a |
| JB14080487/GD/CHN/2008 | HM776269 | 2008 | Guangdong,China | B1a |
| JB14080506/GD/CHN/2008 | HM776270 | 2008 | Guangdong,China | B1a |
| 1172-Yamagata-2008/JPN/2008 | AB634421 | 2008 | Japan | B1a |
| 1316-Yamagata-2008/JPN/2008 | AB634422 | 2008 | Japan | B1a |
| 1386-Yamagata-2008/JPN/2008 | AB634423 | 2008 | Japan | B1a |
| 1694-Yamagata-2008/JPN/2008 | AB634425 | 2008 | Japan | B1a |
| 1768-Yamagata-2008/JPN/2008 | AB634426 | 2008 | Japan | B1a |
| 1817-Yamagata-2008/JPN/2008 | AB634427 | 2008 | Japan | B1a |
| 1892-Yamagata-2008/JPN/2008 | AB634428 | 2008 | Japan | B1a |
| QH0202T/QH/CHN/2008 | GQ429266 | 2008 | Qinghai,China | B1a |
| H135F/SD/CHN/2008/CA16 | GQ253382 | 2008 | Shandong,China | B1a |
| H834F/SD/CHN/2008/CA16 | GQ253386 | 2008 | Shandong,China | B1a |
| ESP08/54682/Spain/2008 | FR798001 | 2008 | Spain | B1a |
| BJ358/BJ/2009/CHN/2009 | JF317970 | 2009 | Beijing,China | B1a |
| BJ388/BJ/CHN/2009 | JF317972 | 2009 | Beijing,China | B1a |
| BJ390/BJ/2009/CHN/2009 | JF317973 | 2009 | Beijing,China | B1a |
| JB141090036/CHN/2009 | HM776272 | 2009 | Guangdong,China | B1a |
| JB141090037/CHN/2009 | HM776273 | 2009 | Guangdong,China | B1a |
| JB141090038/GD/CHN/2009 | HM776274 | 2009 | Guangdong,China | B1a |
| JB141090064/CHN/2009 | HM776275 | 2009 | Guangdong,China | B1a |
| JB142090002/CHN/2009 | HM776276 | 2009 | Guangdong,China | B1a |
| JB142090005/CHN/2009 | HM776277 | 2009 | Guangdong,China | B1a |
| JB142090006/CHN/2009 | HM776278 | 2009 | Guangdong,China | B1a |
| JB143090166/CHN/2009 | HM776284 | 2009 | Guangdong,China | B1a |
| JB143090174/GD/CHN/2009 | HM776285 | 2009 | Guangdong,China | B1a |
| 2077-Yamagata-2009/JPN/2009 | AB634429 | 2009 | Japan | B1a |
| 3350-Yamagata-2009/JPN/2009 | AB634430 | 2009 | Japan | B1a |
| 3351-Yamagata-2009/JPN/2009 | AB634431 | 2009 | Japan | B1a |
| LC0003F/SD/CHN/2009/CA16 | GQ253387 | 2009 | Shandong,China | B1a |
| LC0006F/SD/CHN/2009/CA16 | GQ253388 | 2009 | Shandong,China | B1a |
| LC0017F/SD/CHN/2009/CA16 | GQ253389 | 2009 | Shandong,China | B1a |
| LC0036F/SD/CHN/2009/CA16 | GQ253390 | 2009 | Shandong,China | B1a |
| Ningbo-028/ZJ/2009/CHN/2009 | JQ315095 | 2009 | Zhejiang,China | B1a |
| 2009szk039/ZJ/CHN/2009 | JQ315096 | 2009 | Zhejiang,China | B1a |
| CF160074_FRA10/FRA/2010 | HE572993 | 2010 | France | B1a |
| Nanyang09-2010/HeN/CHN/2010 | JF508448 | 2010 | Henan,China | B1a |
| HN1120/HN/2010/CHN/2010 | JF695002 | 2010 | Henan,China | B1a |
| HN1129/HN/2010/CHN/2010 | JF695003 | 2010 | Henan,China | B1a |
| HN1131/HN/2010/CHN/2010 | JF695004 | 2010 | Henan,China | B1a |
| HN1514/HN/2010/CHN/2010 | JF695005 | 2010 | Henan,China | B1a |
| HN1661/HN/2010/CHN/2010 | JF695008 | 2010 | Henan,China | B1a |
| 925-Yamagata-2010/JPN/2010 | AB634432 | 2010 | Japan | B1a |
| 1043-Yamagata-2010/JPN/2010 | AB634433 | 2010 | Japan | B1a |
| 1173-Yamagata-2010/JPN/2010 | AB634434 | 2010 | Japan | B1a |
| 1636-Yamagata-2010/JPN/2010 | AB634435 | 2010 | Japan | B1a |
| 1984-Yamagata-2010/JPN/2010 | AB634436 | 2010 | Japan | B1a |
| 173-Yamagata-2010/JPN/2010 | AB634437 | 2010 | Japan | B1a |
| 1142-Yamagata-2010/JPN/2010 | AB634438 | 2010 | Japan | B1a |
| 1666-Yamagata-2010/JPN/2010 | AB634439 | 2010 | Japan | B1a |
| 577-Yamagata-2010/JPN/2010 | AB634440 | 2010 | Japan | B1a |
| 820-Yamagata-2010/JPN/2010 | AB634441 | 2010 | Japan | B1a |
| 1160-Yamagata-2010/JPN/2010 | AB634442 | 2010 | Japan | B1a |
| 1289-Yamagata-2010/JPN/2010 | AB634443 | 2010 | Japan | B1a |
| 1306-Yamagata-2010/JPN/2010 | AB634444 | 2010 | Japan | B1a |
| 1448-Yamagata-2010/JPN/2010 | AB634445 | 2010 | Japan | B1a |
| 1327-Yamagata-2010/JPN/2010 | AB634446 | 2010 | Japan | B1a |
| 1375-Yamagata-2010/JPN/2010 | AB634447 | 2010 | Japan | B1a |
| 1575-Yamagata-2010/JPN/2010 | AB634448 | 2010 | Japan | B1a |
| 1700-Yamagata-2010/JPN/2010 | AB634449 | 2010 | Japan | B1a |
| 1876-Yamagata-2010/JPN/2010 | AB634450 | 2010 | Japan | B1a |
| 1973-Yamagata-2010/JPN/2010 | AB634451 | 2010 | Japan | B1a |
| 1931-Yamagata-2010/JPN/2010 | AB634452 | 2010 | Japan | B1a |
| Ningbo-102/ZJ/2010/CHN/2010 | JQ315097 | 2010 | Zhejiang,China | B1a |
| 2010szk017/ZJ/CHN/2010 | JQ315099 | 2010 | Zhejiang,China | B1a |
| 2010Changdao-470/CHN/2010 | JQ315112 | 2010 | Zhejiang,China | B1a |
| SHZH2011-0504/GD/CHN/2011 | JX473415 | 2011 | Guangdong,China | B1a |
| SHZH2011-0505/GD/CHN/2011 | JX473416 | 2011 | Guangdong,China | B1a |
| SHZH2011-0506/GD/CHN/2011 | JX473417 | 2011 | Guangdong,China | B1a |
| SHZH2011-0901/GD/CHN/2011 | JX473425 | 2011 | Guangdong,China | B1a |
| SHZH2011-0902/GD/CHN/2011 | JX473426 | 2011 | Guangdong,China | B1a |
| SHZH2011-1001/GD/CHN/2011 | JX473430 | 2011 | Guangdong,China | B1a |
| SHZH2011-1102/GD/CHN/2011 | JX473436 | 2011 | Guangdong,China | B1a |
| 808-Yamagata-1998/JPN/1998 | AB634324 | 1998 | Japan | B1b |
| Y98-1159/JPN/1998 | AB634321 | 1998 | Japan | B1b |
| 00.108.3206/AUS/2000 | GU142867 | 2000 | Australia | B1b |
| 00.143.2668/AUS/2000 | GU142868 | 2000 | Australia | B1b |
| 188/Toyama/2000/JPN/2000 | AB465372 | 2000 | Japan | B1b |
| 223/Toyama/2000/JPN/2000 | AB465373 | 2000 | Japan | B1b |
| 2055/SA/01 | AM292442 | 2001 | Saudi Arabia | B1b |
| shzh02-111/GD/CHN/2002 | AY895099 | 2002 | Guangdong,China | B1b |
| shzh02-14/GD/CHN/2002 | AY895110 | 2002 | Guangdong,China | B1b |
| shzh02-38/GD/CHN/2002 | AY821795 | 2002 | Guangdong,China | B1b |
| shzh02-75/GD/CHN/2002 | AY895102 | 2002 | Guangdong,China | B1b |
| 124/Toyama/2002/JPN/2002 | AB465374 | 2002 | Japan | B1b |
| 2183-Yamagata-2002/JPN/2002 | AB634383 | 2002 | Japan | B1b |
| 227/Toyama/2002/JPN/2002 | AB465375 | 2002 | Japan | B1b |
| 228/Toyama/2002/JPN/2002 | AB465376 | 2002 | Japan | B1b |
| 246/Toyama/2002/JPN/2002 | AB465377 | 2002 | Japan | B1b |
| 247/Toyama/2002/JPN/2002 | AB465378 | 2002 | Japan | B1b |
| 248/Toyama/2002/JPN/2002 | AB465379 | 2002 | Japan | B1b |
| 249/Toyama/2002/JPN/2002 | AB465380 | 2002 | Japan | B1b |
| 250/Toyama/2002/JPN/2002 | AB465381 | 2002 | Japan | B1b |
| 251/Toyama/2002/JPN/2002 | AB465382 | 2002 | Japan | B1b |
| 252/Toyama/2002/JPN/2002 | AB465383 | 2002 | Japan | B1b |
| 253/Toyama/2002/JPN/2002 | AB465384 | 2002 | Japan | B1b |
| 258/Toyama/2002/JPN/2002 | AB465387 | 2002 | Japan | B1b |
| 260/Toyama/2002/JPN/2002 | AB465389 | 2002 | Japan | B1b |
| 283/Toyama/2002/JPN/2002 | AB465396 | 2002 | Japan | B1b |
| 295/Toyama/2002/JPN/2002 | AB465397 | 2002 | Japan | B1b |
| 298/Toyama/2002/JPN/2002 | AB465398 | 2002 | Japan | B1b |
| 120/Toyama/2003/JPN/2003 | AB465399 | 2003 | Japan | B1b |
| 290/Toyama/2003/JPN/2003 | AB465400 | 2003 | Japan | B1b |
| S110251/SAR/03/MAL/2003 | AM292462 | 2003 | Malaysia | B1b |
| 5338/SA/03 | AM292443 | 2003 | Saudi Arabia | B1b |
| JB140500036/GD/CHN/2005 | HM776221 | 2005 | Guangdong,China | B1b |
| JB140500050/GD/CHN/2005 | HM776222 | 2005 | Guangdong,China | B1b |
| JB140500081/GD/CHN/2005 | HM776225 | 2005 | Guangdong,China | B1b |
| JB140600008/GD/CHN/2006 | HM776228 | 2006 | Guangdong,China | B1b |
| JB140600019/GD/CHN/2006 | HM776229 | 2006 | Guangdong,China | B1b |
| JB140600032/GD/CHN/2006 | HM776232 | 2006 | Guangdong,China | B1b |
| JB140600050/GD/CHN/2006 | HM776235 | 2006 | Guangdong,China | B1b |
| JB140600083/GD/CHN/2006 | HM776243 | 2006 | Guangdong,China | B1b |
| JB140600088/GD/CHN/2006 | HM776245 | 2006 | Guangdong,China | B1b |
| BJ12/BJ/CHN/2007 | JF317961 | 2007 | Beijing，China | B1b |
| BJ26/BJ/CHN/2007 | JF317962 | 2007 | Beijing，China | B1b |
| BJ5/BJ/CHN/2007 | JF317960 | 2007 | Beijing，China | B1b |
| BJ61/BJ/CHN/2007 | JF317963 | 2007 | Beijing，China | B1b |
| 190-D1/FJ/CHN/2007 | JX127258 | 2007 | Fujian,China | B1b |
| GS008T/GS/CHN/2007 | GQ429237 | 2007 | Gansu,China | B1b |
| GS009F/GS/CHN/2007 | GQ429238 | 2007 | Gansu,China | B1b |
| GS011F/GS/CHN/2007 | GQ429239 | 2007 | Gansu,China | B1b |
| JB140700027/GD/CHN/2007 | HM776249 | 2007 | Guangdong,China | B1b |
| NM0710/NM/CHN/2007 | GQ429240 | 2007 | Inner Mongolia,China | B1b |
| 521-01TS/SD/CHN/2007 | GQ429221 | 2007 | Shandong,China | B1b |
| 521-05F/SD/CHN/2007 | GQ429222 | 2007 | Shandong,China | B1b |
| 523-09TS/SD/CHN/2007 | GQ429225 | 2007 | Shandong,China | B1b |
| TC-05F/SD/CHN/2007 | GQ429226 | 2007 | Shandong,China | B1b |
| TC-11F/SD/CHN/2007 | GQ429227 | 2007 | Shandong,China | B1b |
| TC-17F/SD/CHN/2007 | GQ429228 | 2007 | Shandong,China | B1b |
| TC-21F/SD/CHN/2007 | GQ429229 | 2007 | Shandong,China | B1b |
| TC-21F/SD/CHN/2007 | GQ429229 | 2007 | Shandong,China | B1b |
| 00190/Taiwan/CHN/2007 | JF420555 | 2007 | Taiwan,China | B1b |
| BJ115/BJ/CHN/2008 | JF317965 | 2008 | Beijing，China | B1b |
| BJ271/BJ/CHN/2008 | JF317967 | 2008 | Beijing,China | B1b |
| BJ62/BJ/CHN/2008 | JF317964 | 2008 | Beijing,China | B1b |
| GS0030V/GS/CHN/2008 | GQ429243 | 2008 | Gansu,China | B1b |
| GS0191F/GS/CHN/2008 | GQ429248 | 2008 | Gansu,China | B1b |
| GS0271F/GS/CHN/2008 | GQ429250 | 2008 | Gansu,China | B1b |
| GS0276V/GS/CHN/2008 | GQ429251 | 2008 | Gansu,China | B1b |
| GS0278F/GS/CHN/2008 | GQ429252 | 2008 | Gansu,China | B1b |
| GS0279T/GS/CHN/2008 | GQ429253 | 2008 | Gansu,China | B1b |
| GS0281F/GS/CHN/2008 | GQ429254 | 2008 | Gansu,China | B1b |
| GS0365F/GS/CHN/2008 | GQ429256 | 2008 | Gansu,China | B1b |
| GS0366T/GS/CHN/2008 | GQ429257 | 2008 | Gansu,China | B1b |
| GS0393V/GS/CHN/2008 | GQ429259 | 2008 | Gansu,China | B1b |
| GS0405V/GS/CHN/2008 | GQ429260 | 2008 | Gansu,China | B1b |
| GS0417F/GS/CHN/2008 | GQ429261 | 2008 | Gansu,China | B1b |
| JB14080003/GD/CHN/2008 | HM776251 | 2008 | Guangdong,China | B1b |
| JB14080034/GD/CHN/2008 | HM776254 | 2008 | Guangdong,China | B1b |
| JB14080038/GD/CHN/2008 | HM776255 | 2008 | Guangdong,China | B1b |
| JB14080060/GD/CHN/2008 | HM776257 | 2008 | Guangdong,China | B1b |
| JB14080145/GD/CHN/2008 | HM776259 | 2008 | Guangdong,China | B1b |
| JB14080521/GD/CHN/2008 | HM776271 | 2008 | Guangdong,China | B1b |
| 1613-Yamagata-2008/JPN/2008 | AB634424 | 2008 | Japan | B1b |
| QH0189T/QH/CHN/2008 | GQ429264 | 2008 | Qinhai,China | B1b |
| QH0194T/QH/CHN/2008 | GQ429265 | 2008 | Qinhai,China | B1b |
| QH0218T/QH/CHN/2008 | GQ429267 | 2008 | Qinhai,China | B1b |
| QH0269T/QH/CHN/2008 | GQ429268 | 2008 | Qinhai,China | B1b |
| QH0325T/QH/CHN/2008 | GQ429269 | 2008 | Qinhai,China | B1b |
| QH0376T/QH/CHN/2008 | GQ429270 | 2008 | Qinhai,China | B1b |
| QH0377T/QH/CHN/2008 | GQ429271 | 2008 | Qinhai,China | B1b |
| QH0381T/QH/CHN/2008 | GQ429272 | 2008 | Qinhai,China | B1b |
| QH0526T/QH/CHN/2008 | GQ429273 | 2008 | Qinhai,China | B1b |
| QH0549T/QH/CHN/2008 | GQ429274 | 2008 | Qinhai,China | B1b |
| QH0557T/QH/CHN/2008 | GQ429275 | 2008 | Qinhai,China | B1b |
| QH0558T/QH/CHN/2008 | GQ429276 | 2008 | Qinhai,China | B1b |
| QH0570T/QH/CHN/2008 | GQ429277 | 2008 | Qinhai,China | B1b |
| H060F/SD/CHN/2008 | GQ253380 | 2008 | Shandong,China | B1b |
| H113F/SD/CHN/2008 | GQ253381 | 2008 | Shandong,China | B1b |
| H365F/SD/CHN/2008 | GQ253383 | 2008 | Shandong,China | B1b |
| H425F/SD/CHN/2008/CA16 | GQ253384 | 2008 | Shandong,China | B1b |
| H573F/SD/CHN/2008 | GQ253385 | 2008 | Shandong,China | B1b |
| 2008szk290/ZJ/CHN/2008 | JQ315100 | 2008 | Zhejiang,China | B1b |
| Beijing0907/BJ/CHN/2009 | GQ406339 | 2009 | Beijing,China | B1b |
| BJ296/BJ/CHN/2008 | JF317968 | 2009 | Beijing，China | B1b |
| BJ344/BJ/CHN/2009 | JF317969 | 2009 | Beijing，China | B1b |
| BJ359/BJ/CHN/2009 | JF317971 | 2009 | Beijing，China | B1b |
| 15M-E1/FJ/CHN/2009 | JX127255 | 2009 | Fujian,China | B1b |
| 19M-F3/FJ/CHN/2009 | JX127256 | 2009 | Fujian,China | B1b |
| 22M-F1/FJ/CHN/2009 | JX127257 | 2009 | Fujian,China | B1b |
| 3428-E9/FJ/CHN/2009 | JX127261 | 2009 | Fujian,China | B1b |
| 3560-B1/FJ/CHN/2009 | JX127262 | 2009 | Fujian,China | B1b |
| 3583-C6/FJ/CHN/2009 | JX127263 | 2009 | Fujian,China | B1b |
| 3593-A2/FJ/CHN/2009 | JX127264 | 2009 | Fujian,China | B1b |
| 3617-A8/FJ/CHN/2009 | JX127265 | 2009 | Fujian,China | B1b |
| 3629-D1/FJ/CHN/2009 | JX127266 | 2009 | Fujian,China | B1b |
| 3630-D2/FJ/CHN/2009 | JX127267 | 2009 | Fujian,China | B1b |
| 3661-D2/FJ/CHN/2009 | JX127268 | 2009 | Fujian,China | B1b |
| 3669-B8/FJ/CHN/2009 | JX127269 | 2009 | Fujian,China | B1b |
| 3679-D11/FJ/CHN/2009 | JX127270 | 2009 | Fujian,China | B1b |
| 4337-F1/FJ/CHN/2009 | JX127271 | 2009 | Fujian,China | B1b |
| 4380-A11/FJ/CHN/2009 | JX127273 | 2009 | Fujian,China | B1b |
| 4430-A2/FJ/CHN/2009 | JX127274 | 2009 | Fujian,China | B1b |
| 4432-E3/FJ/CHN/2009 | JX127275 | 2009 | Fujian,China | B1b |
| 4456-A6/FJ/CHN/2009 | JX127276 | 2009 | Fujian,China | B1b |
| 4479-B4/FJ/CHN/2009 | JX127277 | 2009 | Fujian,China | B1b |
| E16T/FJ/CHN/2009 | JQ670920 | 2009 | Fujian,China | B1b |
| E4T-F1/FJ/CHN/2009 | JX127284 | 2009 | Fujian,China | B1b |
| XMCDC-0942/FJ/CHN/2009 | JQ320271 | 2009 | Fujian,China | B1b |
| JB143090006/GD/CHN/2009 | HM776279 | 2009 | Guangdong,China | B1b |
| JB143090041/GD/CHN/2009 | HM776280 | 2009 | Guangdong,China | B1b |
| JB143090088/GD/CHN/2009 | HM776281 | 2009 | Guangdong,China | B1b |
| JB143090097/GD/CHN/2009 | HM776282 | 2009 | Guangdong,China | B1b |
| JB143090099/GD/CHN/2009 | HM776283 | 2009 | Guangdong,China | B1b |
| JB143090178/GD/CHN/2009 | HM776286 | 2009 | Guangdong,China | B1b |
| JB143090192/GD/CHN/2009 | HM776287 | 2009 | Guangdong,China | B1b |
| MAS02/AH/CHN/2010 | JQ409498 | 2010 | Henan,China | B1b |
| 213a-A4/FJ/CHN/2010 | JX127259 | 2010 | Fujian,China | B1b |
| 219a-C12/FJ/CHN/2010 | JX127260 | 2010 | Fujian,China | B1b |
| 6737-E2/FJ/CHN/2010 | JX127278 | 2010 | Fujian,China | B1b |
| 6816-D4/FJ/CHN/2010 | JX127279 | 2010 | Fujian,China | B1b |
| 7185-E9/FJ/CHN/2010 | JX127280 | 2010 | Fujian,China | B1b |
| 7195-C5/FJ/CHN/2010 | JX127281 | 2010 | Fujian,China | B1b |
| 7197-D4/FJ/CHN/2010 | JX127282 | 2010 | Fujian,China | B1b |
| 7200-C2/FJ/CHN/2010 | JX127283 | 2010 | Fujian,China | B1b |
| Z49-F5/FJ/CHN/2010 | JX127285 | 2010 | Fujian,China | B1b |
| HN1516/HN/CHN/2010 | JF695006 | 2010 | Henan,China | B1b |
| HN1539/HN/CHN/2010 | JF695007 | 2010 | Henan,China | B1b |
| HN1662/CHN/2010 | JF695009 | 2010 | Henan,China | B1b |
| HN1668/CHN/2010 | JF695010 | 2010 | Henan,China | B1b |
| HN1726/CHN/2010 | JF695011 | 2010 | Henan,China | B1b |
| MAS01/AH/CHN/2010 | JQ409499 | 2010 | Jiangsu,China | B1b |
| MAS03/AH/ CHN/2010 | JQ409497 | 2010 | Jiangsu,China | B1b |
| MAS04/AH/ CHN/2010 | JQ409496 | 2010 | Jiangsu,China | B1b |
| 1903/JL/CHN/2010 | JQ180468 | 2010 | Jilin,China | B1b |
| TJ2010-CA16-08/TJ/CHN/2010 | JX455098 | 2010 | Tianjin,China | B1b |
| TJ2010-CA16-21/TJ/CHN/2010 | JX455099 | 2010 | Tianjin,China | B1b |
| TJ2010-CA16-22/TJ/CHN/2010 | JX455100 | 2010 | Tianjin,China | B1b |
| TJ2010-CA16-38/TJ/CHN/2010 | JX455101 | 2010 | Tianjin,China | B1b |
| 2010Changdao-166/ZJ/CHN/2010 | JQ315104 | 2010 | Zhejiang，China | B1b |
| 2010Changdao-185/ZJ/CHN/2010 | JQ315105 | 2010 | Zhejiang,China | B1b |
| 2010Changdao-191/ZJ/CHN/2010 | JQ315106 | 2010 | Zhejiang，China | B1b |
| 2010Changdao-201/ZJ/CHN/2010 | JQ315107 | 2010 | Zhejiang，China | B1b |
| 2010Changdao-234/ZJ/CHN/2010 | JQ315108 | 2010 | Zhejiang，China | B1b |
| 2010Changdao-412/CHN/2010 | JQ315109 | 2010 | Zhejiang，China | B1b |
| 2010Changdao-439/ZJ/CHN/2010 | JQ315110 | 2010 | Zhejiang，China | B1b |
| 2010Changdao-450/ZJ/CHN/2010 | JQ315111 | 2010 | Zhejiang，China | B1b |
| 2010Changdao-475/ZJ/CHN/2010 | JQ315113 | 2010 | Zhejiang，China | B1b |
| 2010Changdao-477/ZJ/CHN/2010 | JQ315114 | 2010 | Zhejiang,China | B1b |
| 2010Changdao-488/ZJ/CHN/2010 | JQ315115 | 2010 | Zhejiang,China | B1b |
| 2010szk113/ZJ/CHN/2010 | JQ315098 | 2010 | Zhejiang,China | B1b |
| YYS10-044/ZJ/CHN/2010 | JQ315101 | 2010 | Zhejiang,China | B1b |
| YYS10-062/ZJ/CHN/2010 | JQ315102 | 2010 | Zhejiang，China | B1b |
| YYS10-067/ZJ/CHN/2010 | JQ315103 | 2010 | Zhejiang，China | B1b |
| SHZH2011-0501/GD/CHN/2011 | JX473412 | 2011 | Guangdong,China | B1b |
| SHZH2011-0503/GD/CHN/2011 | JX473414 | 2011 | Guangdong,China | B1b |
| SHZH2011-0601/GD/CHN/2011 | JX473418 | 2011 | Guangdong,China | B1b |
| SHZH2011-0701/GD/CHN/2011 | JX473419 | 2011 | Guangdong,China | B1b |
| SHZH2011-0903/GD/CHN/2011 | JX473427 | 2011 | Guangdong,China | B1b |
| SHZH2011-1003/GD/CHN/2011 | JX473432 | 2011 | Guangdong,China | B1b |
| SHZH2011-1004/GD/CHN/2011 | JX473433 | 2011 | Guangdong,China | B1b |
| SHZH2011-1101/GD/CHN/2011 | JX473435 | 2011 | Guangdong,China | B1b |
| SHZH2011-1103/GD/CHN/2011 | JX473437 | 2011 | Guangdong,China | B1b |
| SHZH2011-1104/GD/CHN/2011 | JX473438 | 2011 | Guangdong,China | B1b |
| SX/EV-1/CA16/2011 | JN582179 | 2011 | Shannxi,China | B1b |
| SB16087/SAR/05/MAL/2005 | AM292476 | 2005 | Malaysia | B1c |
| CF279014_FRA10/FRA/2010 | HE572994 | 2010 | France | B1c |
| CF281008_FRA10/FRA/2010 | HE572995 | 2010 | France | B1c |
| CF301047_FRA10/FRA/2010 | HE572996 | 2010 | France | B1c |
| CF312044_FRA10/FRA/2010 | HE572997 | 2010 | France | B1c |
| CF323006_FRA10/FRA/2010 | HE572998 | 2010 | France | B1c |
| CF328001_FRA10/FRA/2010 | HE572999 | 2010 | France | B1c |
| CF335030_FRA10/FRA/2010 | HE573000 | 2010 | France | B1c |
| CF341014_FRA10/FRA/2010 | HE573002 | 2010 | France | B1c |
| CF341020_FRA10/FRA/2010 | HE573003 | 2010 | France | B1c |
| CF348018_FRA10/FRA/2010 | HE573004 | 2010 | France | B1c |
| CF355014_FRA10/FRA/2010 | HE573005 | 2010 | France | B1c |
| CF355015_FRA10/FRA/2010 | HE573006 | 2010 | France | B1c |
| CF361090_FRA10/FRA/2010 | HE573007 | 2010 | France | B1c |
| PM-1795457-07/MAL/2007 | JN248417 | 2007 | Malaysia | B1c |
| PM-31131-05/MAL/2005 | JN248418 | 2005 | Malaysia | B1c |
| PM-1694925-06/MAL/2006 | JN248419 | 2006 | Malaysia | B1c |
| PM-35210-06/MAL/2006 | JN248420 | 2006 | Malaysia | B1c |
| PM-1651402-06/MAL/2006 | JN248421 | 2006 | Malaysia | B1c |
| PM-1824818-07/MAL/2007 | JN248422 | 2007 | Malaysia | B1c |
| 24/Toyama/1981/JPN/1981 | AB465366 | 1981 | Japan | B2 |
| 379/Toyama/1984/JPN/1984 | AB465367 | 1984 | Japan | B2 |
| 576/Toyama/1988/JPN/1988 | AB465368 | 1988 | Japan | B2 |
| 107/Toyama/1990/JPN/1990 | AB465369 | 1990 | Japan | B2 |
| 392/Toyama/1995/JPN/1995 | AB465370 | 1995 | Japan | B2 |
| Y88-5375/JPN/1988 | AB634286 | 1988 | Japan | B2 |
| Y92-2389/JPN/1992 | AB634287 | 1992 | Japan | B2 |
| Y92-2773/JPN/1992 | AB634288 | 1992 | Japan | B2 |
| Y92-2710/JPN/1992 | AB634289 | 1992 | Japan | B2 |
| Y92-2855/JPN/1992 | AB634290 | 1992 | Japan | B2 |
| Y92-2861/JPN/1992 | AB634291 | 1992 | Japan | B2 |
| Y92-2882/JPN/1992 | AB634292 | 1992 | Japan | B2 |
| Y92-2998/JPN/1992 | AB634293 | 1992 | Japan | B2 |
| Y93-2054/JPN/1993 | AB634294 | 1993 | Japan | B2 |
| Y98-891/JPN/1998 | AB634320 | 1998 | Japan | B2 |
| 721-Yamagata-1998/JPN/1998 | AB634322 | 1998 | Japan | B2 |
| 737-Yamagata-1998/JPN/1998 | AB634323 | 1998 | Japan | B2 |
| S10432/SAR/98/MAL/1998 | AM292455 | 1998 | Malaysia | B2 |
| S70382/SAR/98/MAL/1998 | AM292461 | 1998 | Malaysia | B2 |
| SB1660/SAR/00/MAL/2000 | AM292465 | 2000 | Malaysia | B2 |
| SB2239/SAR/00/MAL/2000 | AM292468 | 2000 | Malaysia | B2 |
