# Supplementary material for: Molecular Epidemiology of Coxsackievirus A16: Intratype and Prevalent Intertype Recombination Identified
Source: PLoS One. 2013 Dec 10;8(12):e82861. doi: 10.1371/journal.pone.0082861 (PMC3858299; doi:10.1371/journal.pone.0082861)
Supplement: Table S3 — List of the EV-A prototype strains used for the sequence comparison, phylogenetic and recombination analysis. (DOCX) [file pone.0082861.s003.docx]

**TABLE S3. List of the EV-A prototype strains used for the sequence comparison, phylogenetic and recombination analysis.**

| Abbreviation | Strain name | GenBank Accession Number | The place of isolation | The year of isolation | genotype |
| --- | --- | --- | --- | --- | --- |
| CVA2 | Fleetwood/USA/1947 | AY421760 | USA | 1947 | A |
| CVA3 | Olson/USA/1948 | AY421761 | USA | 1948 | A |
| CVA4 | High Point/USA/1948 | AY421762 | USA | 1948 | A |
| CVA5 | Swartz/USA/1950 | AY421763 | USA | 1950 | A |
| CVA6 | Gdula/USA/1949 | AY421764 | USA | 1949 | A |
| CVA7 | Parker/USA/1949 | AY421765 | USA | 1949 | A |
| CVA8 | Donovan/USA/1949 | AY421766 | USA | 1949 | A |
| CVA10 | Kowalik/USA/1950 | AY421767 | USA | 1950 | A |
| CVA12 | Texas-12/USA/1948 | AY421768 | USA | 1948 | A |
| CVA14 | G-14/SOA/1950 | AY421769 | SOA | 1950 | A |
| CVA16 | G-10/SOA/1951 | U05876 | SOA | 1951 | A |
| EV-A71 | BrCr/USA/1970 | U22521 | USA | 1970 | A |
| EV-A76 | FRA91-10369/FRA/1991 | AY697458 | FRA | 1991 | A |
| EV-A89 | BAN00-10359/BAN/2000 | AY697459 | BAN | 2000 | A |
| EV-A90 | BAN99-10399/BAN/1999 | AY697460 | BAN | 1999 | A |
| EV-A91 | BAN00-10406/USA/1999 | AY697461 | BAN | 1999 | A |
| EV-A92 | RJG7/USA/2007 | EF667344 | USA | 2007 | A |

（J Gen Virol. 2005 Feb;86(Pt 2):445-51. Enteroviruses 76, 89, 90 and 91 represent a novel group within the species Human enterovirus A. Oberste MS, Maher K, Michele SM, Belliot G, Uddin M, Pallansch MA.）
