# Supplementary material for: Molecular Epidemiology of Coxsackievirus A16: Intratype and Prevalent Intertype Recombination Identified
Source: PLoS One. 2013 Dec 10;8(12):e82861. doi: 10.1371/journal.pone.0082861 (PMC3858299; doi:10.1371/journal.pone.0082861)
Supplement: Table S4 — Pairwise nucleotide sequence identities based on corresponding regions of 35 complete genomic sequences of CVA16. (DOCX) [file pone.0082861.s004.docx]

TABLE S4．Pairwise nucleotide sequence identities based on corresponding regions of 35 complete genomic sequences of CVA16

| region | identities (%) | | | |
| --- | --- | --- | --- | --- |
|  | B1a | B1b | B1 | between B1a and B1b |
| complete | 91.4-98.5 | 93.2-98.9 | 89.4-98.9 | 89.4-97.5 |
| 5UTR* | 93.4-99.1 | 95.3-99.1 | 93.2-100 | 93.2-100 |
| P1 | 92.7-98.7 | 93.2-98.8 | 90.3-98.8 | 90.3-96.2 |
| VP4 | 91.3-99.5 | 94.6-100 | 89.8-100 | 89.8-100 |
| VP2 | 91.3-98.8 | 92.3-99.2 | 89.6-99.2 | 89.6-96.9 |
| VP3 | 92.0-99.0 | 92.2-99.4 | 89.3-99.4 | 89.3-95.7 |
| VP1 | 93.1-99.3 | 92.7-99.3 | 89.8-99.3 | 89.8-96.6 |
| P2 | 92.1-98.6 | 93.1-99.0 | 90.5-99.0 | 90.5-99.0 |
| 2A | 90.6-98.6 | 92.2-99.5 | 88.8-99.5 | 88.8-98.6 |
| 2B | 90.3-99.3 | 92.5-99.3 | 90.3-99.3 | 90.5-99.3 |
| 2C | 92.0-99.0 | 92.8-98.9 | 89.8-99.2 | 89.8-99.2 |
| P3 | 92.1-98.5 | 91.3-99.1 | 89.5-99.1 | 89.5-97.5 |
| 3A | 89.5-99.2 | 87.9-99.6 | 84.4-99.6 | 84.4-98.4 |
| 3B | 86.3-100 | 81.8-100 | 81.8-100 | 81.8-95.4 |
| 3C | 89.4-97.9 | 92.3-98.9 | 88.3-98.9 | 88.3-98.9 |
| 3D | 92.7-98.9 | 90.6-99.2 | 89.4-99.2 | 89.4-97.0 |

(*: Pairwise without THA-CA16-090/Thailand/2010, THA-CA16-069/Thailand/2010, HQ09011181/YN/CHN/2011 for partial missing in 5’UTR)
